# Cellular responses to HSV-1 infection are linked to specific types of alterations in the host transcriptome

**DOI:** 10.1038/srep28075

**Published:** 2016-06-29

**Authors:** Benxia Hu, Xin Li, Yongxia Huo, Yafen Yu, Qiuping Zhang, Guijun Chen, Yaping Zhang, Nigel W. Fraser, Dongdong Wu, Jumin Zhou

**Affiliations:** 1 Key Laboratory of Animal Models and Human Disease Mechanisms of the Chinese Academy of Sciences & Yunnan Province, Kunming Institute of Zoology, Kunming, Yunnan 650223, China; 2 Kunming College of Life Science, University of Chinese Academy of Sciences, Kunming, Yunnan 650204, China; 3 School of Life Sciences, Anhui University, Hefei, Anhui 230601, China; 4 State Key Laboratory of Genetic Resources and Evolution, Kunming Institute of Zoology, Chinese Academy of Sciences, Kunming, Yunnan 650223, China; 5 Department of Microbiology, Perelman School of Medicine, University of Pennsylvania, Philadelphia, PA 19104, USA

## Abstract

Pathogen invasion triggers a number of cellular responses and alters the host transcriptome. Here we report that the type of changes to cellular transcriptome is related to the type of cellular functions affected by lytic infection of Herpes Simplex Virus type I in Human primary fibroblasts. Specifically, genes involved in stress responses and nuclear transport exhibited mostly changes in alternative polyadenylation (APA), cell cycle genes showed mostly alternative splicing (AS) changes, while genes in neurogenesis, rarely underwent these changes. Transcriptome wide, the infection resulted in 1,032 cases of AS, 161 incidences of APA, 1,827 events of isoform changes, and up regulation of 596 genes and down regulations of 61 genes compared to uninfected cells. Thus, these findings provided important and specific links between cellular responses to HSV-1 infection and the type of alterations to the host transcriptome, highlighting important roles of RNA processing in virus-host interactions.

Herpes Simplex Virus type I (HSV-1) is a 152 kb double-strand DNA virus containing around 80 genes that infects about 80% of the human population^1^,^2^. Following a primary infection HSV-1 normally enters a latent infection in sensory neurons of periphery sensory ganglions^3^. Stress signals and weakened immunity cause the reactivation of HSV-1 from sensory neurons and lytic infection ensues in epithelial cells which these neurons innervate, resulting in cold sores or Herpes keratitis^4^. HSV-1 lytic infection in cultured cells unfolds rapidly, with the expression of immediate early (IE) genes, including ICP0 and ICP4, the virus quickly recruits host RNA polymerase, transcription co-regulators and chromatin modifying complexes to facilitate viral early gene expression, and prepare for viral DNA synthesis followed by late gene expression, occurring at approximately 6 hours post infection (hpi). The incoming virus triggers a number of host responses including the activation of the interferon pathway^5^,^6^, the DNA damage response^7^,^8^,^9^, apoptosis^10^,^11^ and other host defense mechanisms limiting viral growth^12^. In return, many of the viral genes are designed to modulate these responses to ensure viral transcription, genome synthesis and assembly. ICP34.5, for example, is a key viral factor interfering with the interferon β (IFN-β)pathway^13^,^14^,^15^. ICP27, ICP4 and ICP22, on the other hand, are negative regulators of the host apoptotic response, while ICP8 inhibits the host DNA damage response by inactivating the ATR kinase^16^,^17^,^18^,^19^. ICP0 inhibits host transcription silencing activity by displacing host CoREST silencing complex, while it also degrades RNF8 and RNF168, two ubiquitin ligases in the DDR pathway^8^,^20^,^21^ and components of the PML body^22^. The ICP27 factor also inhibits host RNA splicing^23^,^24^, while the viral host shutoff protein (vhs) degrades host, as well as viral RNAs^25^,^26^,^27^,^28^,^29^.

These viral factors, together with host responses, result in complex viral host interactions, the outcome of which determines whether the virus enters a lytic infection, or becomes suppressed and enters latency. Many details of these processes are reflected in the alterations in the transcriptome of infected host cells. Thus how host cells respond to viral infection at transcriptomic level is an important but under explored question. Studies in HSV-1 infected mouse embryonic fibroblast cells (MEF), mouse cornea and trigeminal ganglion have led to the discovery of new genes and pathways of virus-host interactions^14^,^30^,^31^. However, these studies were done using DNA microarrays with limited genome coverage, thus likely missing many important genes, furthermore these studies are unable to analyze other types of changes such as alternative splicing (AS), alternative polyadenylation (APA) and gene isoform composition.

Transcriptome-sequencing (RNA-seq) has revealed that approximately 94% of human genes are alternatively spliced, generating a much larger diversity of functional variants from a fixed number of genes in the genome^32^. AS is widely known to participate in a wide range of biological processes including virus-host interactions. The HSV-1 encoded ICP27 protein, which exports unspliced mRNA to the cytosol, is also reported to alter PML protein^23^ and glycoprotein C^24^ isoform composition via alternative splicing, while the SM factor from EBV changes isoform expression of STAT for the benefit of EBV infection^33^. At genome wide level, profound changes in AS occur as cells adapt to external stimulus. For example, dendritic cells produced wide spread changes in alternative splicing when encountering bacteria^34^. Also, when compared to normal cells, genome wide changes in splice isoforms were also observed in carcinoma^35^. Although many splicing factors are described, the mechanisms regulating the splicing process in response to external signals are not well understood. The CTCF factor is one of the few known host regulators reported to interact near many intron-exon junctions and alters the rate of RNA Pol II elongation, and favors the splicing of downstream exons. CTCF binding sites are subject to imprinting, thus providing means of epigenetic regulation of alternative splicing^36^.

In addition to AS, more than 50% of human genes are also subject to APA^37^, which changes in response to different physiological conditions or during cell differentiation. For example, increased proliferation, dedifferentiation, and disease conditions are associated with proximal polyadenylation sites, such as C2C12 myoblast compared to C2C12 differentiated myotubes^38^, resting B cell compared to activated B cells^39^, or MCF7 breast cancer cells compared to normal breast epithelial cells^40^. In contrast, when cells exit the cell cycle and become differentiated, distal polyadenylation sites are preferred. Terminally differentiated neurons^41^, and activated B cells are examples^39^. The longer 3′ UTRs tend to contain multiple miRNA targets^42^ and other cis elements^43^, providing fine tuned regulation of gene transcript levels. Although the mechanism is not well understood, the choice of APA sites is affected by the interaction between the C-terminal domain of the largest subunit RNA polymerase II and a processing complex CPSF (cleavage and polyadenylation specificity factor), CstF (cleavage stimulation factor) and the canonical poly(A) signal AAUAAA^44^. It is also affected by the usage of alternative promoters, and by epigenetic modification of local chromatin^44^.

To date there are no reported studies analyzing whether and how viral infection affects host cell RNA splicing or polyadenylation site choices at the transcriptomic level. Here we report an RNA-seq analysis of the HSV-1 infected host transcriptome of human primary fibroblast BJ cells at 6hpi, and found that the infection up regulated 596 genes and down regulated 61 genes, significantly more than previous reports. This analysis identified many new important viral induced genes including epigenetic regulators BRD2 and CBX4, regulator of RNA metabolism such as ZNF36. But, importantly, the infection resulted in 1,032 alternative splicing events, 161 APA and 1,827 cases of gene isoform expression changes. GO analyses revealed that stress response, cell cycle and nuclear transport genes are almost exclusively regulated by AS or APA, genes related to neurogenesis are mostly regulated by differential expression. This pattern suggests important regulatory and mechanistic differences among host responses, and highlights important roles of RNA processing in virus-host interactions.

## Results

Human BJ Skin Fibroblasts cells were used to study human HSV-1 lytic infection. The infection was done with the 17+ strain of HSV-1 at a multiplicity of infection (MOI) of 5 to ensure close to 100% of the cells become infected and the infection is more synchronized among different cells. The infected cells were processed at 6 hpi, and three independent biological repeat samples were pooled for library construction and sequencing. 6 hpi samples reflect an important time point, when viral transcription and replication is active and the host nucleus and host chromatin are still mostly intact, but most genes that are up regulated by HSV-1 infection could be readily detected.

### HSV-1 infection caused differential gene expression in human BJ cells

As the differential expression analysis from RNA-seq data allowed the identification of important new candidate genes, including coding and noncoding genes mediating virus-host interactions. The RNA-seq data consisted of 26 million (26,693,203) 90 bp paired-end reads for infected and similar amount for uninfected control (26,797,247), which was processed by TopHat^45^,^46^ and Cufflinks^47^,^48^,^49^,^50^. Approximately 84% of reads from the control group and 40% from infected sample were aligned to the human genome. The infected sample contained a large amount of paired-end reads aligned to the HSV-1 genome (30%), thus, at 6hpi and 5 MOI, viral RNAs comprise about 40% of the total mapped RNA reads in the infected human BJ cells.

**Figure 1:**
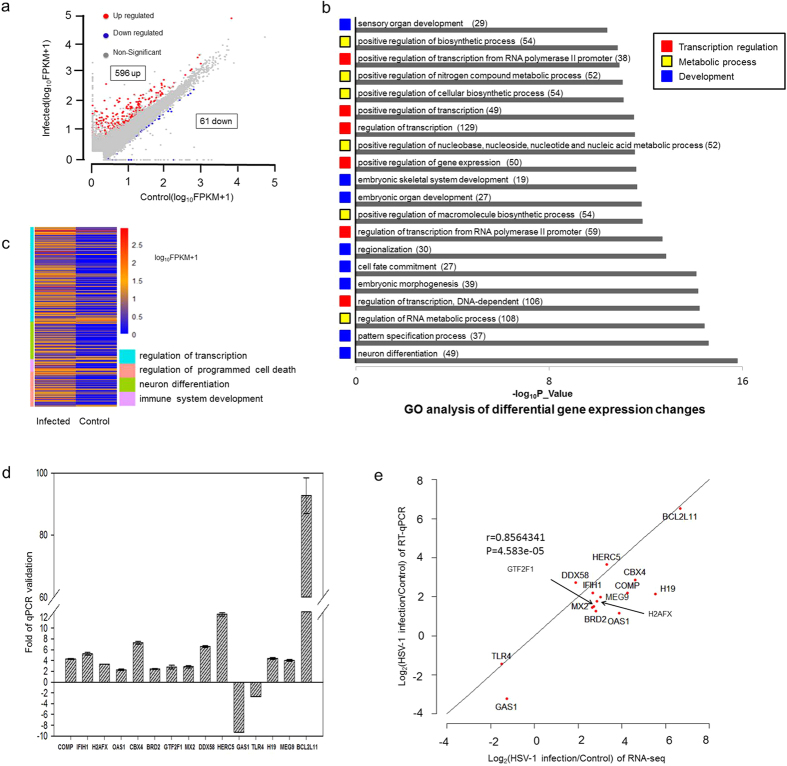
Analyses of differentially expressed genes in HSV-1 infection of BJ cells. Human primary fibroblast BJ cells were infected at 5 moi for 6 hours before being processed for RNA-seq by HI-seq 2000. (**a**) Differentially expressed genes were determined by at least two-fold changes in RNA levels after HSV-1 infection (FPKM value ≥ 1 and a *p*_value ≤ 0.05). A total of 657 genes were differentially expressed after infection including 596 that were up regulated and 61 down regulated. (**b**) Based on *p*_Value, we included the top 20 most significant GO categories affected by HSV-1 infection. Gene numbers in each GO term are indicated in parentheses. Those 20 GO categories were divided into three classes: transcription regulation (marked by red box), metabolic processes (marked by yellow box) and development (marked by blue box). One gene may appear in multiple GO terms. (**c**) The heatmap of differentially expressed genes in four broad categories based on GO analysis: 1) transcriptional regulation, 2) neurogenesis and differentiation, 3) regulation of programed cell death and 4) immune system development in HSV-1 infected and control samples. (**d**) qRT-PCR validation of up-regulated genes CBX4, BRD2, COMP, H2AFX, OAS1, GTF2F1, MX2, DDX58, IFIH1, MEG9, H19, HERC5, BCL2L11 and down-regulated genes GAS1 and TLR4. “Minus” denotes down regulation, “plus” denotes up regulation. All data are normalized to 18S rRNA and presented as means ± stander deviation from three independent biological experiments. (**e**) Correlation analysis between RNA-seq identified genes and qRT-PCR validation.

RNA-seq result showed that the infection led to increased expression of 596 annotated genes, and down-regulation of just 61. Differentially expressed genes are plotted in Fig. 1a. The number of down-regulated genes accounts for only 9.28% of differentially expressed genes, a ratio similar to that of a previous report in infected MEF cells^14^.

Based on gene ontology (GO) analysis differentially expressed genes performed by DAVID^51^, top 20 GO terms from these genes ranked by *p* value are listed in Fig. 1b. Notably, these GO terms could be grouped into three broad categories based on their function, with transcriptional regulation comprised of 6 GO terms (positive regulation of transcription from RNA polymerase II promoter, positive regulation of transcription, regulation of transcription, positive regulation of gene expression, regulation of transcription from RNA polymerase II promoter and regulation of transcription, DNA-dependent), 131 genes, metabolic (mostly nucleic acid, RNA and DNA metabolic) processes has 6 GO terms (positive regulation of biosynthetic process, positive regulation of nitrogen compound metabolic process, positive regulation of cellular biosynthetic process, positive regulation of nucleobase, nucleoside, nucleotide and nucleic acid metabolic process, positive regulation of macromolecule biosynthetic process and regulation of RNA metabolic process), 120 genes, and developmental genes has 8 GO terms (sensory organ development, embryonic skeletal system development, embryonic organ development, regionalization, cell fate commitment, embryonic morphogenesis, pattern specification process and neuron differentiation), 101 genes. These results suggest that transcriptional regulation, metabolic processes and developmental genes are the three most significantly affected classes of genes.

Viral modification of cellular metabolic processes, especially nucleic acid metabolism is crucial for viral transcription, DNA synthesis and alternation of host RNA processing. In contrast, the biological function of activating neurogenic genes by HSV-1 infection is not clear, but this is likely due to the viral ICP0 protein, which displaces the host CoREST repressor complex from silencing viral transcription, in this process ICP0 also derepresses many host neurogenic genes 8,20,21,52.

As shown in the heatmap in Fig. 1c, we extracted the four categories of genes known to be affected by HSV-1 infection from GO analyses result: regulation of transcription (123 genes), neurogenesis (49 genes), apoptosis (46 genes) and immune system development (17 genes) to group the differentially expressed genes (listed in Supplementary Table S1). The infection affected genes in these categories are similar to a previous analyses in MEF cells^14^, but containing many newly identified genes (listed in Supplementary Table S2), suggesting that the RNA-seq used in the present analysis revealed changes in the virus infected transcriptome in much greater details.

Transcriptional regulation may play a key role in driving many cellular processes in response to viral infection. For example, two key epigenetic regulators, BRD2^53^,^54^ and CBX4^55^,^56^,^57^ are highly induced genes (Fig. 1d,e), possibly to reprogram the cellular epigenetic landscape in respond to the infection, or to facilitate viral transcription.

Apoptosis is known to be induced and modified by HSV infection^14^. BCL2L11, an important factor in apoptosis pathway^58^,^59^,^60^, was up regulated 103 fold (Fig. 1d,e), suggesting increased apoptotic signaling as a result of cellular stress. TP73 is another pro-apoptotic gene activated by the DNA damage response^61^. It was up regulated by 81-fold. In contrast, as the virus takes over the control of the host cells, it also inhibits the apoptotic process to allow the virus to reproduce, or to establish latent infection. For example, PIM3, a Serine/Threonine kinase family of proto-oncogene with an anti-apoptotic property^62^,^63^, was up regulated 8-fold by HSV-1 infection (Supplementary Table S1).

HSV-1 infection is known to induce DNA damage response (DDR) and recruits DNA repair factors to the viral replication centers^64^. Here we found three genes H2AFX (Fig. 1d, e), XAB2 (Supplementary Fig. S1) and PAPD7 were up regulated by the infection. H2AFX is the precursor of modified histone γH2A.X, a key chromatin mark of DNA damage response (DDR)^65^,^66^, XAB2 is involved in transcription-coupled repair (TCR)^67^,^68^, while PAPD7 is a DNA polymerase involved in DNA repair and sister chromatid cohesion^69^. The induction of these genes suggests that HSV-1 infection activated DDR may also lead to gene expression level changes in DDR associated factors.

Like all viral infections, host inflammatory reaction (cytokines and chemokines) and intrinsic antiviral response genes (the interferon pathway) are activated. A table of HSV-1 infection induced inflammatory genes and interferon β (IFN β) regulated genes are listed in Supplementary Table S1, Table S2. During infection, HSV-1 also modulates the host interferon response through ICP34.5 and many commonly activated immunity genes are attenuated over time^14^. Several examples of newly identified innate immunity genes include DDX58 (DEAD Box Polypeptide 58), OAS1 (2′-5′-Oligoadenylate Synthetase 1) and TLR4 (Toll-Like Receptor 4) (Fig. 1d,e). DDX58 (also known as RIG-I), a cellular viral sensor^70^,^71^, was activated 6.6 fold by the infection. OAS1, an interferon activated 2-5A synthetase^72^,^73^, which synthesizes 2′,5′-oligoadenylates to activate RNase L to degrade viral (as well as cellular) RNAs, was activated 2.2 fold by HSV-1 infection. In contrast, TLR4, a member of the Toll-Like Receptor family of cellular membrane viral sensors^74^,^75^, was down regulated 2.6 fold by the infection. These examples highlighted the activation or modulation of cellular antiviral response by the incoming virus.

In addition to protein coding genes, we also discovered 51 Long noncoding RNAs which were significantly regulated after HSV-1 infection, including H19 and MEG9 (Fig. 1d,e). We subsequently selected several newly identified host genes that are either up or down regulated following infection for qRT-PCR validation. qRT-PCR results exhibited the similar gene expression level changes as the RNA-seq analysis, with a correlation coefficient, *r* of 0.85 (*p* value < 0.01, Pearson test), thus validating the analysis (Fig. 1d,e, see primer information in Supplementary Table S3).

### HSV-1 infection induced host alternative splicing

**Figure 2:**
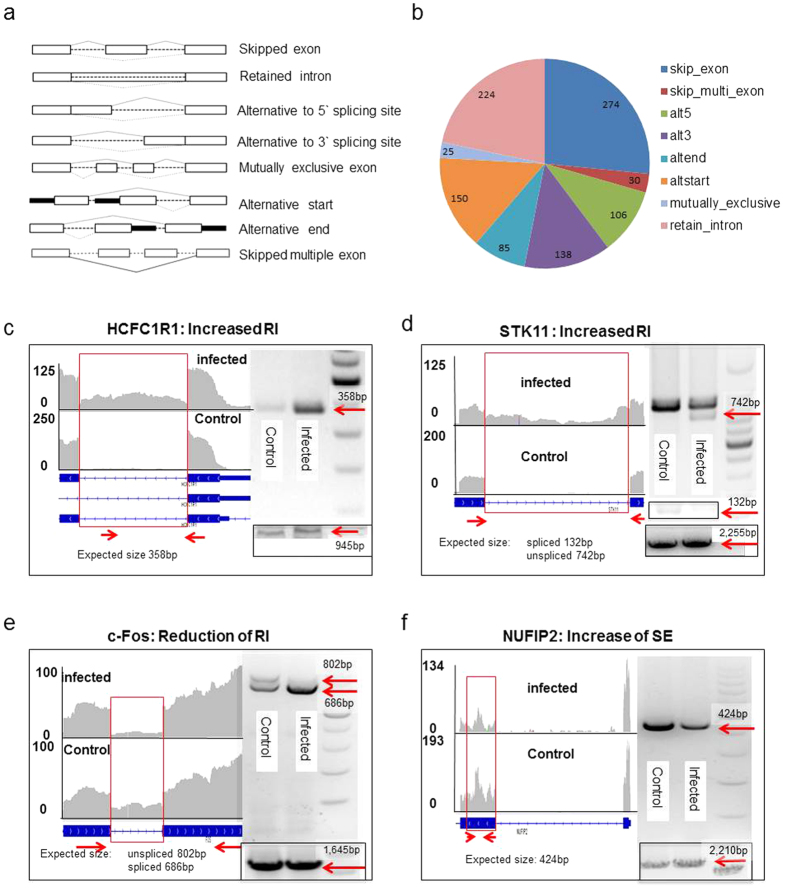
HSV-1 infection induced alternative splicing of the cellular transcriptome. (**a**) Eight types of alternative splicing^32^,^76^. (**b**) 1,032 significant alternative splicing genes were detected, which falls into skipped exon, skipped multiple exon, alternative to 5′ splicing site (AS5), alternative to 3′ splicing site (AS3), alternative start (altstart), alternative end (altend), mutually exclusive and retained intron classes. (**c**–**f**) Visualization by IGV of RNA sequencing reads and RT-PCR validation of alternative splicing. Y-axis shows the number of mapped reads. The black box in the bottom right corner showed an extended fragment amplified product as a RT-PCR loading control. **c**. HCFC1R1: increased RI after infection is shown by the increased intronic PCR product of 358bp, The 945bp fragment is a leading control to show the overall gene expression level. (**d**) STK11: increased RI is demonstrated by the reduction of a 132 bp fragment from the spliced form of STK11. In the infected samples, there is a 742 bp unspliced form appearing. The 2,255 bp is a control PCR fragment of this gene to show loading and expression level of this gene, (**e**) c-Fos: the PCR primers amplify a unspliced fragment of 802 bp, but an spliced form of 686 bp. The 1,645 bp fragment if an internal control to show the total RNA level (spliced and unspliced) of c-FOS. (**f**) NUFIP2: the PCR product (424 bp) is from the exon exhibiting skipped exon (SE). The 2,210 fragment is a control PCR to show an extended region of this gene, which is unchanged after the infection.

Alternative splicing (AS) events^32^,^76^ include skipped exon (SE), retained intron (RI), alternative to 5′ splicing site (AS5), alternative to 3′ splicing site (AS3), mutually exclusive exon (MXE), alternative start (altstart), alternative end (altend) and skip multiple exons (skip_multi_exon) (see Fig. 2a for a graphic explanation). We used ASD software^76^ to analyze the RNA-seq data, and found 1,032 significant AS events occurred in 883 genes after HSV-1 infection. While most AS events (771) happened once per gene, they occurred twice in 80 genes, three times in 25 genes, four times in 4 genes (ARNTL, ATXN2L, MAP3K12 and U2AF1L4) and five times in 2 genes (B4GALT4 and FHOD1). The occurrence of different types of AS is summarized in Fig. 2b. Notably, the SE type of AS (274 cases) comprising the largest share, over a quarter, of all AS events. The second largest group, 21.7% of all AS events, is intron retention (RI).

To confirm this analysis, we validated 4 AS events in 4 genes, HCFC1R1 (host cell factor C1 regulator 1), STK11 (Serine/Threonine Kinase 11), c-Fos and NUFIP2 (Nuclear fragile X mental retardation-interacting protein 2), using primers designed to detect the changes (Supplementary Table S3). In Fig. 2c,d, both HCFC1R1 and STK11 showed more retained introns after infection, as RT-PCR using primers specifically amplifying the introns in question detected an increase in amount of fragment size of 358 bp and 742 bp for these two genes, respectively, confirming the increase of unspliced intron (arrows in Fig. 2c,d).

In contrast, c-Fos, an important immediate early cellular gene, which senses stress^77^, showed increased splicing after infection (Fig. 2e). Normally, the c-Fos transcript is highly unstable with a half-life of only 15 minutes. Its RNA is only partially spliced with the unspliced precursor RNA quickly degraded due to a destabilizing element present in the third intron^77^,^78^. Stress signals promote the splicing and produce stable c-Fos mRNA. From the RNA-seq result, the third intron became completely spliced after the infection, along with a significant increase of the more stable c-Fos mRNA. Using primers that could distinguish spliced (686 bp: arrow in Fig. 2e) and unspliced intron number 3 (116 bp) we detected two fragments of 802 bp and 686 bp with similar intensity before infection, but detected mostly the 686 bp following infection (Fig. 2e). This result confirmed the increased splicing of intron number 3 in the c-Fos gene, suggesting that HSV-1 infection activated the cellular stress response by increasing the splicing of c-Fos pre mRNA. An example of SE type of alternative splicing is seen in the NUFIP2 gene. To verify this, we used primers specific to detect the skipped exon and the RT-PCR result showed a drop in the amount of 424 bp fragment after infection, indicating exon skipping (Fig. 2f).

### HSV-1 infection led to changes in polyadenylation in the host transcriptome

**Figure 3:**
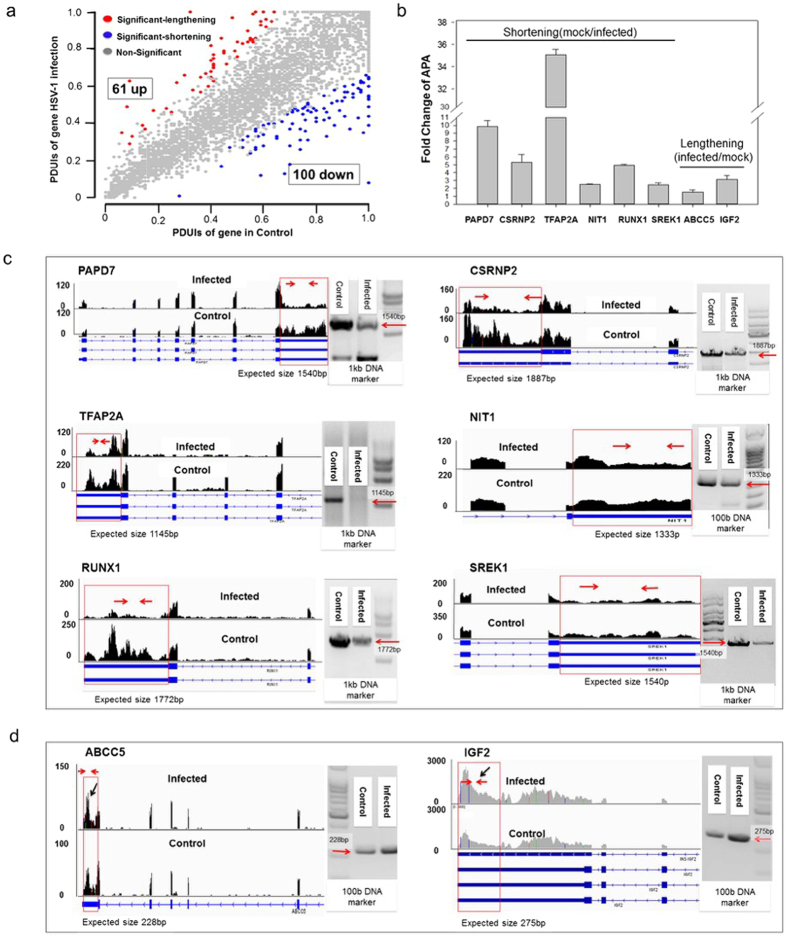
HSV-1 infection resulted in changes in the alternative polyadenylation profile of the host transcriptome. (**a**) A total of 161 cases of changes in APA, including 61 lengthening and 100 of shortening of gene 3′ UTRs. PDUI is a unit measure of distal polyadenylation site usage, a large PDUI value indicates higher distal site usage. (**b**) RT-qPCR validation of the 3′UTR of 6 genes: PAPD7, CSRNP2, TFAP2A, NIT1, RUNX1 and SREK1. All data are normalized to 18S rRNA and presented as means ± stander deviation from three independent biological experiments. Ordinate shows fold changes of control/infected sample (shortening) or fold changes of infected/control (lengthing). (**c**) Visualization by IGV of RNA sequencing reads and RT-PCR validation of these 6 APA genes using primers that best revealed the differences. Y-axis shows the number of mapped reads. (**d**) Two example of increase in the length of a 3′UTR. Y-axis shows the number of mapped reads.

The 3′ UTRs of genes often contain miRNA targets^42^ and other cis elements^43^, which could modulate the turnover of mRNA. For genes with multiple polyadenylation sites, the selection of a proximal or a distal site could be an important regulatory mechanism to control gene expression. Thus we analyzed the changes in host APA pattern using DaPars^79^ software to identify de novo dynamic APAs from RNA-seq data, and found a total of 161 significant cases of APA changes, with 61 cases displaying a shift from proximal polyadenylation sites to distal ones, resulting in longer 3′ UTRs, and 100 cases showed switching from distal polyadenylation sites to proximal sites, leading to shorter 3′ UTRs (Fig. 3a, Supplementary Table S4). As shortening of the 3′UTRs is masked to a certain degree by existing, longer UTRs from the same genes prior to the infection, the 100 cases are likely an underestimate of the actual 3′ UTR shortening events. Thus, the overall changes of APA pattern are biased toward shortening of the 3′UTR, which, together with the increase of skipped exons (Fig. 2b), suggests that HSV-1 infection led to an overall shortening of affected gene transcripts.

We then chose 6 genes that exhibited shortening of 3′ UTR after the infection and conducted qRT-PCR and RT-PCR (primer information in Supplementary Table S3) experiments to validate the analysis. SREK1^80^ is a RNA processing factor, CSRNP2^81^ and TFAP2A^82^ are transcription activators, PAPD7^69^,^83^ and NIT1 are DNA damage response factor^84^, and RUNX1 is a cell immunity factor^85^. As shown in Fig. 3b, qRT-PCR validation of the 3′UTR of these genes showed reduction of regions of the 3′UTRs specific to the longer form, changes consistent with the APA analysis using the DaPars software^79^. The RT-PCR validation of these genes is shown in Fig. 3c. Here, the PAPD7 gene showed an increase of reads in the last exon, but a drop in the 3′ UTR (boxed area), indicating a shortening. RT-PCR result showed a reduction of a predicted 1,540bp product. Similar results were seen in the remainder 5 genes, while HSV-1 infection slightly reduced the reads of the exons of these genes, the 3′ UTR regions were significantly and specifically reduced compared to the rest of these genes (see Fig. 3c boxed areas). Thus, both qRT-PCR and RT-PCR analyses showed changes consistent with RNA-seq analysis, confirming the increased usage of proximal APA sites. In contrast, two examples of the lengthening of 3′UTR are shown in Fig. 3d. Here the 3′ reads corresponding to the longer form of the UTR of both ABCC5 and IGF2 genes showed increases (see arrows in Fig. 3d and qRT-PCR validation in Fig. 3b).

### HSV-1 infection induced wide spread changes in isoform composition in the host transcriptome

**Figure 4:**
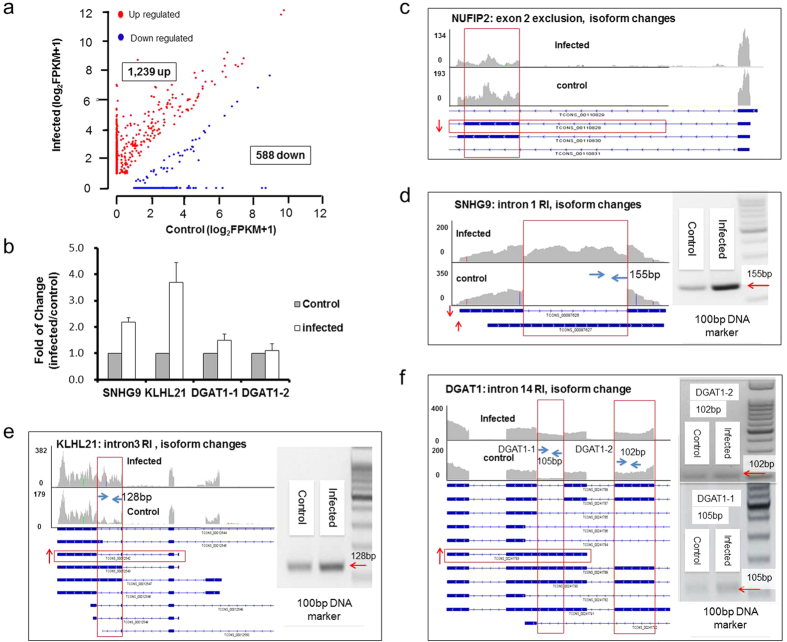
Isoform changes in HSV-1 infected BJ cell transcriptome. (**a**) Scatterplot of log2(FPKM+1) values for isoform changes in non-infected samples (x-axis) and infected samples (y-axis). Genes with isoform changes of at least 2-fold (FPKM ≥ 1, *p*_value ≤ 0.05) was plotted. A total of 1,827 significant isoform changes were found with 1,239 gene isoforms showing increase of expression (red dots) and 588 showing reduction (blue dots) after infection. (**b**) qRT-PCR validation of SNHG9, KLHL21 and DGAT1 genes changes after infection. Two primer pairs (DGAT1-1 and DGAT1-2) were used to determine the change of DGAT1 isoform 00241783. The DGAT1-1 primer can identify 00241783, 00241789, 00241790 and 00241791 isoforms. The DGAT1-2 primer can identify 00241787, 00241788, 00241789, 00241790 and 00241791 isoforms. By comparing the products of DGAT1-1 and DGAT1-2 primer pairs, we can see there is a real increase of isoform 00241783 after infection other than a combined effect of serval isoforms. All data are normalized to 18S rRNA and presented as means ± standard deviation from three independent biological experiments. (**c–f**) Examples of genes with isoform changes. Arrows indicate either the increase or decrease of specific isoforms. Vertical boxed area showed increase of reads after the infection. RT-PCR validation of NUFIP2 showed in Fig. 2f, others showed beside RNA-seq visualization data respectively. Y-axis shows the number of mapped reads.

Isoform composition is an important aspect of the cellular transcriptome and reflects different cellular states and cell identity. Using TopHat^45^,^46^ and Cufflinks^47^,^48^,^49^,^50^ softwares, we found 1,239 cases of increase in isoform expression and 588 cases of descrease in isoform expression after HSV-1 infection (plotted in Fig. 4a). These changes occurred in 1,674 genes, with 1,544 genes containing changes in one isoform, 114 genes showing changes in two isoforms, 11 genes with three isoform changes, 4 genes with 4 isoform changes, and 1 gene with changes in 6 isoforms.

Examples of isoform-specific expression changes are shown in Fig. 4b–f. SNHG9, a long intergenic noncoding RNA, has 2 isoforms, 00087626 and 00087627, the infection resulted in the significant expression of isoform 00087627 (Fig. 4b,d, note the boxed area). As the 00087626 isoform is a spliced form of 00087627, this infection induced isoform switch is also a result of retained intron. The NUFIP2 gene contains 4 isoforms, with the infection reduced 00110828 specific reads (Fig. 4c, red boxed area). KLHL21 has 9 isoforms, HSV-1 infection increased the expression of transcript 00012543, which could be seen from the vertical boxed area where reads specific to isoform 00012543 showed a robust increase (Fig. 4b,e). Finally, DGAT1 also has 11 isoforms and HSV-1 infection induced the expression of 00241783, as evidenced by the appearance of reads inside the red vertical box (Fig. 4b,f).

**Figure 5:**
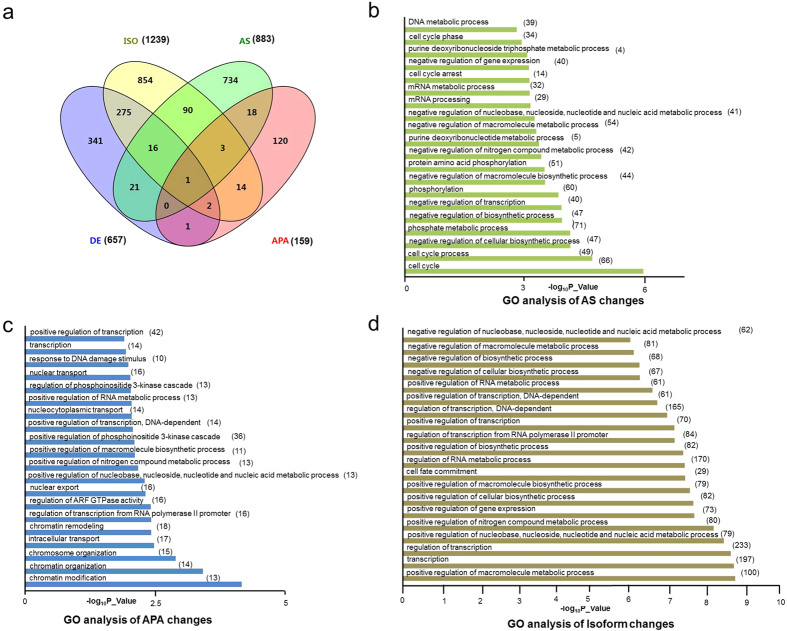
GO analyses of AS, APA and isoform changes in HSV-1 infected cells. (**a**) The overlap of the affected genes of differentially expressed genes (DE), gene isoform changes (ISO), alternative splicing (AS) and alternative polyadenylation (APA). The number in brackets indicated gene number refers to each type. (**b–d**) List of top 20 most significant GO categories in alternative splicing (**b**), APA changes (**c**) and isoform changes (**d**) changes due to HSV-1 infection. Gene numbers in each GO term are indicated in parentheses.

To determine how changes in isoform composition is related to that in differentiall gene expression, we compared 1,255 annotated genes that showed isoform composition changes with 657 annotated, differentially expressed genes and found 294 cases (or 23.43%) overlap, indicating a significantly large propotion of isoform composition changes (76.57%) did not result in gene expression changes (Fig. 5a). In most of the 294 cases examined, the changes of isoform level were in the same direction of the changes of gene expression, however, we found 12 cases where isoform expression level changes happened in the opposite directions with gene expression level changes, suggesting that these genes were subject to mutiple levels of regulation. This result revealed that gene isoform changes were more prevalent than differential gene expression, and in most cases, these changes did not lead to significant changes in overall gene expression levels in viral infected host transcriptome.

When genes with changes in AS and isoform compositon were compared, we found only 110 genes with AS changes also had changes in isoform compositions (Fig. 5a). This is far fewer than expected, as alternative splicing is believed to be a major cause of isoform composition change. However, similar results have been observed by others^86^,^87^, suggesting that other mechanisms, for example, alternative promoter usage or RNA turnover, may contribute more to changes in the composition of gene isoforms. We also compared genes with isoform changes and genes with APA changes, and found only 20 cases of overlap, a small fraction of the total isoform changes, as APA is not significantly related to gene isoform composition changes (Fig. 5a). Finally, comparison of AS with APA genes revealed 22 cases of overlap, suggesting again that APA is an independently regulated event and does not contribute significantly to AS (Fig. 5a).

### GO analysis links cellular responses to viral infection to the types of alteration in the host transcriptome

As described earlier in Fig. 1b, we performed GO analysis to reveal the type of biological processes exhibiting changes in AS, APA and isoform expression after HSV-1 infection. The top 20 significant GO terms in differential gene expression, AS, APA and isoform changes are listed in Fig. 1b and Fig. 5b–d. The GO terms for alternative splicing changes are concentrated in cell cycle (4 terms, 67 genes), metabolic and catabolic processes (12 terms, 187 genes), and transcription (2 GO terms, 40 genes) (Fig. 5b, Supplementary Table S5), suggesting that AS is an important mechanism regulating these processes, which may quickly generate functionally different variants in response to HSV-1 infection. In the APA GO terms (Fig. 5c and Supplementary Table S4), chromatin and transcription regulation occupy the largest shares with 8 GO terms, 32 genes, among these most displayed 3′UTR shortening. The remainder, nuclear transport has 2 terms, 13 genes, metabolic process has 4 terms, 13 genes as did stress responses with 13 genes, suggesting that processing the 3′ UTR played an important role in these cellular processes in response to viral infection.

In the isoform change category (Fig. 5d), 12 of the 20 GO terms including 235 genes are involved in metabolic, especially nucleic acid metabolic pathways, while 7 GO terms with 247 genes are involved in transcription, suggesting that isoform expression switch is a key regulatory mechanism for these two processes in the host cells after viral infection. Finally, as described earlier (Fig. 1b) in differential gene expression analysis, we found that the largest group of GO terms belongs to neurogenesis and development process (8 GO terms, 101 genes), but these terms are notably missing from AS, APA and isoform analyses, again suggesting that these genes are primarily regulated by changing the amount of transcript, not at the level of splicing.

**Figure 6:**
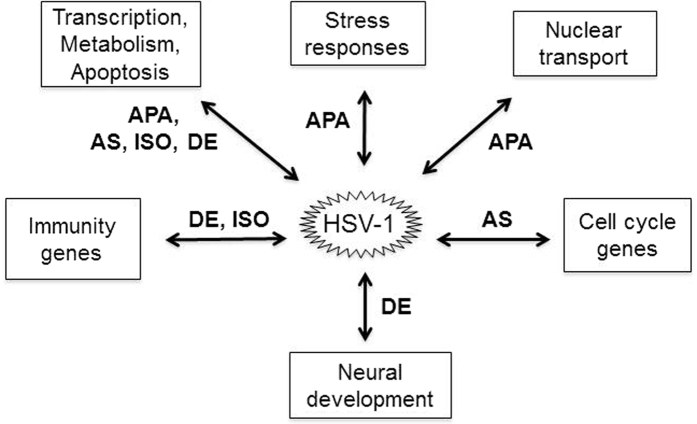


We next combined the similar GO terms into six main cellular processes, 1) transcription and chromatin, 2) cell cycle, 3) metabolic processes, 4) nuclear transport, 5) neurogenesis and 6) stress response, and compared the changes in AS, APA, isoform composition and differential gene expression in each of these processes (Table 1). We found that genes in transcription and chromatin, and metabolic process employ all 4 types of regulation. However, the genes in the remaining GO terms are affected mostly through one specific type. For example, HSV-1 infection induced cell cycle genes underwent only in AS changes (see a list in Supplementary Table S5), while nuclear transport genes and stress responses genes were mostly regulated by APA (also see Supplementary Table S4). Two main host responses, immunity and apoptosis are also significant but are not within the top 20 for each type of transcriptomic changes. We found 25 immunity genes underwent isoform switch and 17 genes exhibited differential gene expression after viral infection (Supplementary Table S6). None of immunity genes has AS or APA changes. In contrast, apoptotic genes have undergone all four types of changes with 81 genes having changes in gene isoform compositions, 30 in differential gene expression, 52 in AS and 16 in APA, respectively (see list in Supplementary Table S6). Taken together, these results strongly suggest mechanistic links between viral infection activated cellular pathways and specific types of alterations in the host transcriptome (Fig. 6).

## Discussion

HSV-1 lytic infection triggers a cascade of cellular events and responses, its effects on cellular gene expression and function are the product of complex virus-host interactions. Although many details of HSV productive infection are known, our understanding of its effects on the host transcriptome is very limited. In an attempt to obtain insights into the mechanism of virus-host interactions, Kent *et al*.^30^ examined latently infected mouse trigeminal ganglia by microarray and found that HSV-1 induced 56 differentially expressed genes, including neuronal-specific genes and several signaling molecules that could promote the initiation of HSV-1 infection. Pasieka *et al*.^14^ detected the change of host cell gene expression also through microarray in HSV-1 infected mouse MEF cells, and found 347 up regulated and 19 down regulated genes. Pasieka *et al*.^31^ performed array analysis of HSV-1 infected mouse corneas and observed roles for viral vhs and the host STAT signaling pathway in viral host interactions. These analyses, while providing valuable and important mechanistic clues to host responses and virus-host interactions, are of limited scope due to the coverage of DNA arrays used. To gain a more comprehensive picture of the host transcriptomic changes in HSV-1 infected human cells, we performed RNA-seq analysis of infected human primary fibroblast BJ cells, and found a significantly larger number of differentially expressed genes in infected cells when compared to previous DNA array studies. The newly identified genes include epigenetic regulators, components of the DDR, regulators of apoptotic pathway, and genes mediating the immune response. For example, CBX4 and BRD2 are previously unreported genes that were highly up regulated, suggesting that these epigenetic regulators may play important roles in the viral infected transcriptome. Overall, the RNA-seq analysis revealed many more cases of increase than decrease of gene expression following HSV-1 infection, an observation similar to Pasieka *et al*.^14^, which is likely due to the fact that existing RNAs prior to the infection masked the decrease.

In a recent study, Rutkowski *et al*., analyzed nascent transcripts in HSV-1 infected human epithelial cells, and found that the infection induced wide spread read through of RNA polymerase resulting in fortuitous expression of hundreds of downstream genes^88^. Thus, to a certain degree, a portion of the infection-induced transcriptome changes noted in our RNA-seq analyses of thousands of genes may not serve relevant biological functions.

Importantly, our RNA-seq analysis reveals detailed changes in host RNA splicing, 3′UTR length and gene isoform composition after the infection. Together we found 1,032 cases of AS changes, encompassing skipped exons, retained introns, alternative to 5′ or 3′ splicing site, mutually exclusive exon, alternative start, alternative end and skip multiple exons (Fig. 2b). We suspect that many of the SE cases observed here were due to HSV-1 infection induced activation of DNA damage response, as skipped exons were seen in cells under genotoxic stress^77^. Indeed, many of the cell cycle genes underwent AS are of the SE type, CENPE and ZC3HC1 are two examples. A notable case highlighting the importance of AS is the c-Fos gene, which becomes more spliced as a result of infection, producing a more stable mRNA, and thus serves as a mechanism of activating the stress response^77^, How viral infection led to changes of AS is an important yet little understood question. The viral factor ICP27 is known to disrupt host splicing, resulting in more unspliced mRNAs^23^,^24^. Indeed, we found 224 cases, or 21.7% of the changes in AS belong to retained introns, and presumably, many of the increases in intron retention were due to the activity of ICP27. However, the remaining AS events are most likely the result of regulatory activities other than that of ICP27. For example, our analysis detected 304 cases (29.5%) of skipped exons or multiple skipped exons following HSV-1 infection (Fig. 2b). Part of these is likely due to an HSV-1 infection induced DDR, which is known to cause skipped exons^89^.

HSV-1 infection also elicited a change in the 3′ UTR through APA. In a total of 161 cases, we found 100 resulted in shortening, suggesting that the majority of affected genes shifted towards making shorter transcripts under viral infection. For example, two stress response genes, SSRP1, PCBP4, (and 6 other validated examples of proximal polyadenylation site usage (Fig. 3b,c) after the infection produced shorter transcripts. In addition to AS and APA, the HSV-1 infected cell transcriptome showed profound changes in gene isoform composition, including 1,239 cases of increases in isoform expression and 588 cases of descrease in isoform expression occurring in 1,674 genes (Fig. 4a). Taken together, the infection resulted in 657 differential expressed genes, but 2,149 genes with AS, APA and isoform changes combined (Fig. 5a), suggesting that the regulation of the host transcriptome at the level of RNA processing is more extensive, and plays at least as important a role in virus-host interactions, as regulation at the transcription level.

More importantly, our analysis revealed a pattern suggesting that HSV-1 induced cellular responses or pathways that are linked to specific types of changes to the host transcriptome (Table 1 and Fig. 6). For example, cell cycle related genes are almost exclusively regulated at the level of AS after infection (listed in Supplementary Table S5), stress response genes are regulated at the level of APA (also see a list in Supplementary Table S4), neural specific genes are regulated mostly by differential expression, while immunity genes are regulated at the level of differential expression and isoform composition changes, and nuclear transport genes are regulated both by AS and APA. In contrast, genes involved in transcription and chromatin and genes in metabolic, mostly nucleic acid, RNA and DNA metabolic processes displayed all four types of changes after the infection. Although, the finding that viral ICP0 protein induced derepression of neuronal genes and genotoxic stress induced changes of AS in cell cycle genes offer some clues as to why cellular responses to viral infection are linked to specific patterns of transcriptome changes, the underlying mechanisms are likely complex and are mostly unknown.

Here we observed that genes involved in RNA metabolism underwent the most profound changes, which include differential gene expression, AS, APA and isoform composition changes. From differentially expressed genes alone, we recovered 108 genes involved in RNA metabolism, which may offer candidates connecting cellular pathways to transcriptomic changes. Particularly, we found up-regulation of several genes that are specifically involved in splicing regulation (Supplementary Fig. S1). For example, PABPC1 is known to participate in AS and APA regulation^90^. YBX1 mediates pre-mRNA alternative splicing regulation^91^,^92^. XAB2 is involved in transcription-coupled repair (TCR), transcription and pre-mRNA splicing^67^,^68^, while, ZFP36^93^,^94^ is also an RNA splicing regulator. Thus, this analysis offers an interesting opportunity to further investigate mechanism of HSV-1 infection induced regulation of RNA processing, and host transcriptomic changes. Future studies in this direction should shed light on how cellular processes affect the transcriptome.

## Materials and Methods

The methods were carried out in accordance with the approved guidelines.

### Cells and virus

Human BJ Skin Fibroblasts cells (ATCC CRL-2522) were purchased from ATCC (Manassas, VA) and grown in complete Dulbecco’s modified Eagle’s medium (DMEM, Life technology Gibco, USA) containing 10% fetal bovine serum (FBS, Gibco, USA), 1% penicillin/streptomycin (Gibco, USA) in a humidified 5% CO2 atmosphere at 37 °C. HSV-1 stain 17+ was used in this study. Virus was grown and titrated on Vero cells (ATCC, USA). Viral infections were done according to standard protocols. Briefly, cultured cells were replaced with serum free DMEM, followed by adding the virus and incubating for 1 hour with occasional rotation to get an even spread, then the culture medium was replaced by regular DMEM with 10% FBS and 1% antibiotics. All experiments were carried out in accordance with the approved guidelines of ethics committee of Kunming Institute of Zoology, and all experimental protocol were approved by ethics committee of Kunming Institute of Zoology, Chinese Academy of Sciences.

### RNA extraction and sequencing

BJ cells were infected with HSV-1 at an MOI of 5 and harvested at 6 hours post infection. The total RNA was extracted with TRIzol reagent (Ambion, 15596-018) following the manufacturer’s instructions. As HSV-1 infection is a rapid process, sequencing individual infections would lead to high level of variation. To circumvent this issue, we pooled the RNA samples from three biological repeats. After the DNase I treatment, magnetic beads with Oligo (dT) were used to isolate mRNA, and then mixed with a fragmentation buffer to degrade the mRNA into shorter fragments. Next, cDNA is synthesized using the mRNA fragments as templates. Short fragments are purified and resolved with EB buffer for sticky ends repair and single nucleotide A addition. After that, the short fragments are connected with adapters. After agarose gel electrophoresis, the fragments averaging 200 bp) are selected for the PCR amplification as templates. During the quality control steps, an Agilent 2100 Bioanaylzer and ABI StepOnePlus Real-Time PCR System were used in quantification and qualification of the sample library. Finally, the library was sequenced using Illumina HiSeqTM 2000.

### RT-PCR validation

BJ cells were infected with HSV-1 at an MOI of 5 and harvested at 6 hours post infection. The total RNA was extracted with TRIzol reagent (Ambion, 15596-018) following the manufacturer’s instructions. One microgram of RNA was subjected to a DNase treatment with RQ1 RNase-Free DNase (Promega). cDNA derived from this RNA was synthesized using RevertAid H Minus First Strand cDNA Synthesis Kit and Random Hexamer Primer. The synthesized cDNAs were amplified with TaKaRa PCR Amplification Kit. Primers used to amplify each gene are listed in supplementary Table S3b. The products were analyzed using gel electrophoresis.

### RT-qPCR validation

1 μg RNA was reverse transcribed using a primeScript RT and DNA Eraser (TaKaRa, DRR047A) Reagent Kits and stored at −80 °C. Real time PCR was run in triplicate with 50 ng cDNA using FastStart Universal SYBR Green Master (Roche, 04913914001) and ABI7900HT. Relative differences were determined using the ΔΔCt approach. ΔΔCt = (Ct_infection_−Ct_18S rRNA_)−(Ct_unfection_−Ct_18S rRNA_). The fold enrichment value is 2^−ΔΔCt^.

### Data analysis for RNA-seq data: Gene and Isoform differential expression analysis

Clean paired-end RNA-seq reads with 90 bp in each end were aligned to the human genome (Homo sapiens (release 37.72), Homo_sapiens.GRCh37.72.gtf) using the TopHat program (v2.0.9) with default parameters. Cufflinks (v2.1.1) was used to calculate Gene and Isoform expression level. We used cutoff (*p*_Value≤ 0.05, FPKM (fragments per kilobaseof exon per million fragments mapped) ≥1 at least one group; FPKM fold ≥2) to select significant, differentially expressed genes and isoforms at 6hpi. We used Tophat (v2.0.9) to align the remainder of unmapped paired-end reads to the HSV-1 strain 17 plus genome (GenBank: JN555585.1).

### Alternative splicing analysis

The “accepted_hits.bam” files generated by Tophat (v2.0.9) were used to detect alternative splicing using the Java program, ASD (AS detector) (v1.2) with the annotation file Homo_sapiens.GRCh37.72.gtf. We selected significant alternatively splicing cases with adjusted_*p*_Value ≤ 0.05.

### Alternative polyadenylation analysis

Clean paired-end RNA-seq reads were aligned to the human genome (Hg19) using TopHat (v2.0.9). We used DaPars (Dynamic analysis of Alternative PolyAdenylation from RNA-seq) to identify APA with default parameters (hg19 bed file). PDUI is a unit measure of distal polyadenylation site usage (dPAS)^79^, a large PDUI value indicates higher distal site usage. To quantify the relative PAS (poly A site) usage, Dapars defined the percentage of dPAS usage for each sample as PDUI index^79^. The greater the PDUI is, the more the dPAS of a transcript is used and vice versa. We selected significant APA with following criterion: FDR ≤ 0.05, |ΔPDUI| = |PDUI_infected_ − PDUI_control_| ≥ 0.2, |log_2_(PDUI_infected_/PDUI_control_)| ≥ 1.5.

### Gene Ontology analysis

We separately uploaded differential expression genes/AS genes/APA genes/Isoform genes into DAVID. DAVID calculated a *p* value for gene enrichment with a modified Fisher’s exact test, and a Benjamin-Hochberg multiple test correction. We selected significant GO terms with *p*_value ≤ 0.05.

### Statistical analysis

We used R relative packages, such as pheatmap (pheatmap: Pretty Heatmaps, Raivo Kolde, 2015) and VennDiagram (VennDiagram: Generate High-Resolution Venn and Euler Plots, Hanbo Chen, 2015), and functions, such as cor.test() to analyze data and draw figures.

## Additional Information

**How to cite this article**: Hu, B. *et al*. Cellular responses to HSV-1 infection are linked to specific types of alterations in the host transcriptome. *Sci. Rep.* **6**, 28075; doi: 10.1038/srep28075 (2016).

## Supplementary Material

Supplementary Information

## Figures and Tables

**Table 1:** GO term distribution in AS, APA, gene isoform and differential expression changes in the transcriptome.

GO term	Type of Change			
	AS	APA	ISO	DE
Transcription and chromatin	2	8	7	6
Cell cycle	4	0	0	0
Metabolic processes	12	4	12	6
Nuclear transport	0	2	0	0
Neurogenesis	0	0	0	8
Stress response	0	4	0	0

The number of GO terms from each broad category (transcription and chromatin, cell cycle, metabolic processes, nuclear transport, neurogenesis, and stress response) are allocated among four types of changes in the host transcriptome: Alternative Splicing (AS), Alternative Polyadenylation (APA), Gene Isoform changes (ISO) and Differential Expression (DE) of the host transcriptome.
