# Supplementary material for: Cellular responses to HSV-1 infection are linked to specific types of alterations in the host transcriptome
Source: Sci Rep. 2016 Jun 29;6:28075. doi: 10.1038/srep28075 (PMC4926211; doi:10.1038/srep28075)
Supplement: Supplementary Information [file srep28075-s1.pdf]

**Supplemental Materials**

# Cellular responses to HSV-1 infection are linked to specific types of alterations in the host transcriptome

Benxia Hu*[1,2,3]; Xin Li*[1,2]; Yongxia Huo[1]; Yafen Yu[1,3]; Qiuping Zhang[1]; Guijun Chen[1]; Yaping Zhang[4]; Nigel W. Fraser[5]; Dongdong Wu[4,6] and Jumin Zhou[1,6]

[1] Key Laboratory of Animal Models and Human Disease Mechanisms of the Chinese Academy of Sciences & Yunnan Province, Kunming Institute of Zoology, Kunming, Yunnan 650223, China.

[2] Kunming College of Life Science, University of Chinese Academy of Sciences, Kunming, Yunnan 650204, China.

[3] School of Life Sciences, Anhui University, Hefei, Anhui 230601, China.

[4] State Key Laboratory of Genetic Resources and Evolution, Kunming Institute of Zoology, Chinese Academy of Sciences, Kunming, Yunnan 650223, China.

[5] Department of Microbiology, Perelman School of Medicine, University of Pennsylvania, Philadelphia, PA 19104, USA.

[6] Correspondence and requests for materials should be addressed to J.Z. (email: zhoujm@mail.kiz.ac.cn) or D.W. (email: wudongdong@mail.kiz.ac.cn).

* These authors contributed equally to the manuscript.

**Table S1. Lists of differentially expressed genes in BJ cells at 6 hours after HSV-1 infection**

**Table S1a.** Genes involved in transcription regulation

| gene | HSV-1 | Control | *p*_value | up/down | fold |
|---|---|---|---|---|---|
| WT1 | 1.14171 | 0 | 0.0003 | up | #DIV/0! |
| UNCX | 20.6764 | 0 | 0.00005 | up | #DIV/0! |
| TLX3 | 1.92401 | 0 | 0.00085 | up | #DIV/0! |
| SOX3 | 7.17119 | 0 | 0.00005 | up | #DIV/0! |
| SOX17 | 2.28655 | 0 | 0.00005 | up | #DIV/0! |
| SHH | 2.79388 | 0 | 0.00005 | up | #DIV/0! |
| SCRT1 | 3.05899 | 0 | 0.00055 | up | #DIV/0! |
| SALL3 | 1.21138 | 0 | 0.00005 | up | #DIV/0! |
| PTF1A | 1.05502 | 0 | 0.01725 | up | #DIV/0! |
| POU3F3 | 1.3903 | 0 | 0.00005 | up | #DIV/0! |
| PAX2 | 1.0963 | 0 | 0.0001 | up | #DIV/0! |
| OVOL1 | 3.51891 | 0 | 0.00005 | up | #DIV/0! |
| OLIG2 | 3.65011 | 0 | 0.00005 | up | #DIV/0! |
| NKX2-6 | 2.01181 | 0 | 0.00005 | up | #DIV/0! |
| NKX2-3 | 2.18119 | 0 | 0.00005 | up | #DIV/0! |
| NKX2-2 | 1.91895 | 0 | 0.00015 | up | #DIV/0! |
| NKX1-1 | 2.70977 | 0 | 0.00005 | up | #DIV/0! |
| MYOD1 | 1.18339 | 0 | 0.0009 | up | #DIV/0! |
| MNX1 | 2.73041 | 0 | 0.00005 | up | #DIV/0! |
| MAFA | 28.7823 | 0 | 0.00005 | up | #DIV/0! |
| LHX5 | 3.29605 | 0 | 0.00005 | up | #DIV/0! |
| LHX1 | 20.3244 | 0 | 0.00005 | up | #DIV/0! |
| LBX1 | 2.72728 | 0 | 0.00005 | up | #DIV/0! |
| IRX4 | 1.28543 | 0 | 0.0006 | up | #DIV/0! |
| IRF4 | 11.7536 | 0 | 0.00005 | up | #DIV/0! |
| INSM2 | 1.35514 | 0 | 0.00005 | up | #DIV/0! |
| INSM1 | 18.894 | 0 | 0.00005 | up | #DIV/0! |
| IHH | 3.14161 | 0 | 0.00005 | up | #DIV/0! |
| HOXD1 | 1.68629 | 0 | 0.00065 | up | #DIV/0! |
| HMX2 | 2.09533 | 0 | 0.0004 | up | #DIV/0! |
| HMX1 | 1.05907 | 0 | 0.0006 | up | #DIV/0! |
| HELT | 1.48308 | 0 | 0.00355 | up | #DIV/0! |
| FOXI3 | 2.76671 | 0 | 0.00005 | up | #DIV/0! |
| FOXD3 | 4.88129 | 0 | 0.00005 | up | #DIV/0! |
| FEZF2 | 2.56052 | 0 | 0.00005 | up | #DIV/0! |
| EMX1 | 1.2298 | 0 | 0.00815 | up | #DIV/0! |
| BHLHE23 | 1.92407 | 0 | 0.00005 | up | #DIV/0! |
| BARX1 | 1.04328 | 0 | 0.01845 | up | #DIV/0! |

| | | | | | |
|---|---|---|---|---|---|
| BARHL2 | 1.48562 | 0 | 0.0002 | up | #DIV/0! |
| ABLIM2 | 1.06455 | 0 | 0.0001 | up | #DIV/0! |
| POU3F3 | 1.3903 | 0 | 0.00005 | up | #DIV/0! |
| RASD1 | 427.64 | 1.34478 | 0.00225 | up | 318.0000 |
| PAX6 | 6.60228 | 0.070558 | 0.0119 | up | 93.57185 |
| TP73 | 1.73934 | 0.02143 | 0.00005 | up | 81.16379 |
| LHX2 | 2.73358 | 0.048131 | 0.03875 | up | 56.79423 |
| SIM2 | 4.76614 | 0.084379 | 0.01105 | up | 56.48511 |
| GRIN1 | 6.11188 | 0.131014 | 0.00115 | up | 46.65059 |
| MAFB | 11.1928 | 0.262338 | 0.0314 | up | 42.66557 |
| ZNF296 | 57.3836 | 1.39528 | 0.009 | up | 41.12694 |
| FOXA1 | 1.12097 | 0.028054 | 0.02195 | up | 39.95758 |
| SIK1 | 16.0143 | 0.43474 | 0.00335 | up | 36.8365 |
| HOXB3 | 4.33852 | 0.142719 | 0.00005 | up | 30.39904 |
| HOXB4 | 4.33852 | 0.142719 | 0.00005 | up | 30.39904 |
| HOXB5 | 4.33852 | 0.142719 | 0.00005 | up | 30.39904 |
| HOXB6 | 4.33852 | 0.142719 | 0.00005 | up | 30.39904 |
| TBX1 | 3.1919 | 0.111056 | 0.04565 | up | 28.74136 |
| HES6 | 17.0382 | 0.674175 | 0.0282 | up | 25.27267 |
| CBX4 | 105.055 | 4.29997 | 0.00005 | up | 24.43157 |
| EGR2 | 2.44428 | 0.13837 | 0.027 | up | 17.66481 |
| HOXA5 | 40.6655 | 2.53485 | 0.00115 | up | 16.04257 |
| HOXA3 | 40.6655 | 2.53485 | 0.00115 | up | 16.04257 |
| HOXA4 | 40.6655 | 2.53485 | 0.00115 | up | 16.04257 |
| HOXA6 | 40.6655 | 2.53485 | 0.00115 | up | 16.04257 |
| DLX1 | 7.15192 | 0.531305 | 0.04065 | up | 13.46104 |
| DLX2 | 18.7771 | 1.47386 | 0.00835 | up | 12.74008 |
| IRX5 | 17.7871 | 1.68531 | 0.00285 | up | 10.5542 |
| ID4 | 11.6723 | 1.13829 | 0.0383 | up | 10.25424 |
| GDNF | 18.2254 | 2.24611 | 0.00065 | up | 8.114206 |
| IRX2 | 13.2151 | 1.64879 | 0.0063 | up | 8.015029 |
| JUNB | 180.492 | 23.4482 | 0.002 | up | 7.697478 |
| MSX1 | 45.7906 | 5.95609 | 0.00035 | up | 7.68803 |
| ATF3 | 14.6006 | 1.89943 | 0.0037 | up | 7.686832 |
| NR4A3 | 4.64376 | 0.604134 | 0.01035 | up | 7.686639 |
| TUBB2B | 41.56 | 5.46301 | 0.0012 | up | 7.607528 |
| UBTF | 133.269 | 17.8362 | 0.0005 | up | 7.471827 |
| RUNX3 | 2.2623 | 0.304667 | 0.0337 | up | 7.425484 |
| SATB2 | 11.5165 | 1.64256 | 0.00055 | up | 7.011312 |
| GTF2F1 | 247.6 | 39.7922 | 0.0001 | up | 6.222325 |
| ZNF503 | 15.7326 | 2.68675 | 0.0034 | up | 5.855625 |
| ING1 | 32.1321 | 5.502 | 0.0274 | up | 5.840076 |
| KLF4 | 17.5026 | 3.02304 | 0.003 | up | 5.789735 |

| | | | | | |
|---|---|---|---|---|---|
| ZNF347 | 14.898 | 2.66475 | 0.02185 | up | 5.590768 |
| FOXC2 | 16.1969 | 3.06861 | 0.00155 | up | 5.278253 |
| ALDH1A3 | 19.0468 | 3.67015 | 0.03105 | up | 5.189652 |
| STAT4 | 1.79078 | 0.375829 | 0.04765 | up | 4.76488 |
| SOX9 | 8.88515 | 1.95518 | 0.0072 | up | 4.544415 |
| NFATC2 | 1.99964 | 0.44037 | 0.0386 | up | 4.540818 |
| BCORL1 | 11.5295 | 2.54563 | 0.00155 | up | 4.529134 |
| GATA2 | 122.683 | 27.5001 | 0.0008 | up | 4.461184 |
| NOTCH1 | 11.5316 | 2.74678 | 0.0011 | up | 4.198225 |
| MAF | 9.58315 | 2.29626 | 0.0095 | up | 4.173373 |
| ISL2 | 8.02966 | 1.93784 | 0.03795 | up | 4.143614 |
| SNAPC4 | 11.6576 | 2.82657 | 0.00255 | up | 4.124292 |
| KLF2 | 37.896 | 9.21056 | 0.0023 | up | 4.114408 |
| FOXQ1 | 14.545 | 3.60684 | 0.0367 | up | 4.032616 |
| POLR2A | 134.722 | 35.1064 | 0.0003 | up | 3.837534 |
| KDM6B | 9.52747 | 2.57009 | 0.0399 | up | 3.707057 |
| MBTD1 | 8.26336 | 2.25114 | 0.01475 | up | 3.670745 |
| IRF7 | 11.0408 | 3.06429 | 0.04375 | up | 3.603053 |
| TCF7 | 11.1386 | 3.1029 | 0.0073 | up | 3.589739 |
| ERN1 | 15.8008 | 4.44049 | 0.01565 | up | 3.558346 |
| FOXC1 | 15.0835 | 4.4641 | 0.01085 | up | 3.378845 |
| IRF1 | 19.9638 | 6.21186 | 0.0315 | up | 3.21382 |
| NFATC1 | 5.88226 | 1.85807 | 0.0467 | up | 3.16579 |
| CDK11A | 38.9246 | 12.5399 | 0.00975 | up | 3.10406 |
| SLC35E2 | 38.9246 | 12.5399 | 0.00975 | up | 3.10406 |
| GLI3 | 2.61378 | 0.856528 | 0.04375 | up | 3.051599 |
| ZNF408 | 8.69526 | 2.87942 | 0.0339 | up | 3.019796 |
| FOS | 51.8218 | 17.6804 | 0.03005 | up | 2.931031 |
| VEGFA | 38.8331 | 14.0268 | 0.00875 | up | 2.768493 |
| SAFB2 | 45.2718 | 16.3879 | 0.02955 | up | 2.762514 |
| TAF3 | 9.58737 | 3.51305 | 0.0224 | up | 2.729073 |
| POU4F3 | 14.8763 | 5.53615 | 0.01785 | up | 2.68712 |
| RBM27 | 14.8763 | 5.53615 | 0.01785 | up | 2.68712 |
| SKI | 44.9982 | 16.7995 | 0.0072 | up | 2.678544 |
| CXXC1 | 32.459 | 12.3814 | 0.02685 | up | 2.621594 |
| HNRNPAB | 186.523 | 73.1812 | 0.0483 | up | 2.548783 |
| YBX1 | 872.446 | 352.301 | 0.0286 | up | 2.476422 |
| EGR1 | 39.4461 | 16.4595 | 0.0224 | up | 2.396555 |
| MLL5 | 22.5226 | 9.40712 | 0.0247 | up | 2.394208 |
| HOXA10 | 110.895 | 47.9207 | 0.03065 | up | 2.314136 |
| HOXA9 | 110.895 | 47.9207 | 0.03065 | up | 2.314136 |
| SHOX | 79.6494 | 34.6435 | 0.0212 | up | 2.299115 |
| SETD1B | 7.88346 | 3.50085 | 0.03375 | up | 2.25187 |

| | | | | | |
|---|---|---|---|---|---|
| SERTAD1 | 37.7325 | 16.7702 | 0.04095 | up | 2.249973 |
| ZNF573 | 0.592931 | 3.54229 | 0.0322 | down | 5.974203 |
| TLR4 | 3.29112 | 9.34058 | 0.03035 | down | 2.838116 |
| ZNF84 | 1.80156 | 4.59739 | 0.03715 | down | 2.551894 |
| RGMB | 48.0976 | 117.243 | 0.02085 | down | 2.437606 |
| MAML2 | 3.02629 | 6.5995 | 0.04785 | down | 2.180723 |
| ZNF146 | 10.241 | 22.2416 | 0.04745 | down | 2.171819 |
| TAF7 | 22.9733 | 48.7069 | 0.04505 | down | 2.120153 |

**Table S1b.** Genes involved in neuronal differentiation

| gene | HSV-1 | Control | *p*_value | up/down | fold |
|---|---|---|---|---|---|
| TLX3 | 1.92401 | 0 | 0.00085 | up | #DIV/0! |
| SHH | 2.79388 | 0 | 5.00E-05 | up | #DIV/0! |
| SALL3 | 1.21138 | 0 | 5.00E-05 | up | #DIV/0! |
| PTF1A | 1.05502 | 0 | 0.01725 | up | #DIV/0! |
| PAX2 | 1.0963 | 0 | 0.0001 | up | #DIV/0! |
| OLIG2 | 3.65011 | 0 | 5.00E-05 | up | #DIV/0! |
| NKX2-2 | 1.91895 | 0 | 0.00015 | up | #DIV/0! |
| LHX5 | 3.29605 | 0 | 5.00E-05 | up | #DIV/0! |
| LHX1 | 20.3244 | 0 | 5.00E-05 | up | #DIV/0! |
| LBX1 | 2.72728 | 0 | 5.00E-05 | up | #DIV/0! |
| HELT | 1.48308 | 0 | 0.00355 | up | #DIV/0! |
| FEZF2 | 2.56052 | 0 | 5.00E-05 | up | #DIV/0! |
| EMX1 | 1.2298 | 0 | 0.00815 | up | #DIV/0! |
| BARHL2 | 1.48562 | 0 | 0.0002 | up | #DIV/0! |
| CNTN2 | 1.12529 | 0.00467001 | 0.03695 | up | 240.9609401 |
| PAX6 | 6.60228 | 0.0705584 | 0.0119 | up | 93.5718497 |
| TP73 | 1.73934 | 0.02143 | 5.00E-05 | up | 81.16378908 |
| LHX2 | 2.73358 | 0.0481313 | 0.03875 | up | 56.79422746 |
| FOXA1 | 1.12097 | 0.028054 | 0.02195 | up | 39.95758181 |
| EGR2 | 2.44428 | 0.13837 | 0.027 | up | 17.66481174 |
| RTN4R | 8.65676 | 0.617656 | 0.01835 | up | 14.01550378 |
| DLX1 | 7.15192 | 0.531305 | 0.04065 | up | 13.46104403 |
| DLX2 | 18.7771 | 1.47386 | 0.00835 | up | 12.74008386 |
| EPHA4 | 25.3012 | 2.35923 | 5.00E-05 | up | 10.7243465 |
| IRX5 | 17.7871 | 1.68531 | 0.00285 | up | 10.55420071 |
| ID4 | 11.6723 | 1.13829 | 0.0383 | up | 10.25424101 |
| JAG1 | 5.93672 | 0.649868 | 0.018 | up | 9.135270547 |
| CDK5R1 | 4.59189 | 0.538381 | 0.0127 | up | 8.52907142 |
| GDNF | 18.2254 | 2.24611 | 0.00065 | up | 8.114206339 |
| SEMA6C | 8.90094 | 1.13409 | 0.02385 | up | 7.84853054 |

| | | | | | |
|---|---|---|---|---|---|
| TUBB2B | 41.56 | 5.46301 | 0.0012 | up | 7.607527718 |
| RUNX3 | 2.2623 | 0.304667 | 0.0337 | up | 7.425484217 |
| MX1 | 32.1865 | 6.17649 | 0.0021 | up | 5.211131241 |
| SEMA6A | 4.64885 | 0.921882 | 0.01195 | up | 5.042782048 |
| GATA2 | 122.683 | 27.5001 | 0.0008 | up | 4.461183778 |
| DFNB31 | 5.18905 | 1.2215 | 0.0454 | up | 4.248096603 |
| NOTCH1 | 11.5316 | 2.74678 | 0.0011 | up | 4.198224831 |
| ISL2 | 8.02966 | 1.93784 | 0.03795 | up | 4.143613508 |
| EFNB1 | 14.4019 | 4.4855 | 0.01675 | up | 3.21076803 |
| GLI3 | 2.61378 | 0.856528 | 0.04375 | up | 3.051599014 |
| VEGFA | 38.8331 | 14.0268 | 0.00875 | up | 2.76849317 |
| POU4F3 | 14.8763 | 5.53615 | 1.79E-02 | up | 2.687120111 |
| RBM27 | 14.8763 | 5.53615 | 1.79E-02 | up | 2.687120111 |
| TUBB2A | 105.963 | 39.4337 | 0.00665 | up | 2.687117871 |
| OPHN1 | 9.79002 | 3.89637 | 0.0337 | up | 2.512600189 |
| EPHA2 | 40.7057 | 19.997 | 0.04285 | up | 2.035590339 |
| PTPRR | 0.0982346 | 1.51339 | 0.01325 | down | 15.40587532 |
| NTNG1 | 5.78403 | 14.7999 | 0.0174 | down | 2.558752289 |
| GAS1 | 8.53322 | 20.3735 | 0.04705 | down | 2.387551241 |
| SLIT2 | 15.7407 | 33.7294 | 0.02785 | down | 2.142814487 |

**Table S1c.** Genes involved in immune system development

| gene | HSV-1 | Control | *p*_value | up/down | fold |
|---|---|---|---|---|---|
| IRF4 | 11.7536 | 0 | 0.00005 | up | #DIV/0! |
| BARX1 | 1.04328 | 0 | 0.01845 | up | #DIV/0! |
| NKX2-3 | 2.18119 | 0 | 0.00005 | up | #DIV/0! |
| BCL2L11 | 114.119 | 1.10871 | 0.00005 | up | 102.9295307 |
| HOXB3 | 4.33852 | 0.142719 | 0.00005 | up | 30.39904 |
| HOXB4 | 4.33852 | 0.142719 | 0.00005 | up | 30.39904 |
| HOXB5 | 4.33852 | 0.142719 | 0.00005 | up | 30.39904 |
| HOXB6 | 4.33852 | 0.142719 | 0.00005 | up | 30.39904 |
| TBX1 | 3.1919 | 0.111056 | 0.04565 | up | 28.74135571 |
| HOXA3 | 40.6655 | 2.53485 | 0.00115 | up | 16.04256662 |
| HOXA4 | 40.6655 | 2.53485 | 0.00115 | up | 16.04256662 |
| HOXA5 | 40.6655 | 2.53485 | 0.00115 | up | 16.04256662 |
| HOXA6 | 40.6655 | 2.53485 | 0.00115 | up | 16.04256662 |
| PLCG2 | 2.25892 | 0.199476 | 0.0001 | up | 11.32426959 |
| JAG1 | 5.93672 | 0.649868 | 0.018 | up | 9.135270547 |
| ACE | 10.7536 | 2.05749 | 0.00335 | up | 5.226562462 |
| VEGFA | 38.8331 | 14.0268 | 0.00875 | up | 2.76849317 |
| EGR1 | 39.4461 | 16.4595 | 0.0224 | up | 2.396555181 |

| | | | | | |
|---|---|---|---|---|---|
| MLL5 | 22.5226 | 9.40712 | 0.0247 | up | 2.394207792 |
| HOXA10 | 110.895 | 47.9207 | 0.03065 | up | 2.314135645 |
| HOXA9 | 110.895 | 47.9207 | 0.03065 | up | 2.314135645 |
| KITLG | 15.2164 | 34.0663 | 0.033 | down | 2.238788413 |

**Table S1d.** Genes involved in programmed cell death regulation

| gene | HSV-1 | Control | *p*_value | up/down | fold |
|---|---|---|---|---|---|
| SHH | 2.79388 | 0 | 0.00005 | up | #DIV/0! |
| PROC | 2.28778 | 0 | 0.00005 | up | #DIV/0! |
| POU3F3 | 1.3903 | 0 | 0.00005 | up | #DIV/0! |
| PAX2 | 1.0963 | 0 | 0.0001 | up | #DIV/0! |
| NKX2-6 | 2.01181 | 0 | 0.00005 | up | #DIV/0! |
| MUC5AC | 3.42509 | 0 | 0.00005 | up | #DIV/0! |
| MUC2 | 2.78512 | 0 | 0.00005 | up | #DIV/0! |
| IHH | 3.14161 | 0 | 0.00005 | up | #DIV/0! |
| CRYAA | 5.12522 | 0 | 0.00005 | up | #DIV/0! |
| BHLHE23 | 1.92407 | 0 | 0.00005 | up | #DIV/0! |
| ACTN2 | 1.32554 | 0 | 0.0042 | up | #DIV/0! |
| FGF4 | 1.28328 | 0 | 0.00005 | up | #DIV/0! |
| BCL2L11 | 114.119 | 1.10871 | 0.00005 | up | 102.9295 |
| TP73 | 1.73934 | 0.02143 | 0.00005 | up | 81.16379 |
| GRIN1 | 6.11188 | 0.131014 | 0.00115 | up | 46.65059 |
| CBX4 | 105.055 | 4.29997 | 0.00005 | up | 24.43157 |
| COMP | 30.6532 | 1.60156 | 0.0041 | up | 19.13959 |
| COL2A1 | 1.83852 | 0.102768 | 0.0281 | up | 17.89 |
| DLX1 | 7.15192 | 0.531305 | 0.04065 | up | 13.46104 |
| PLCG2 | 2.25892 | 0.199476 | 0.0001 | up | 11.32427 |
| CDK5R1 | 4.59189 | 0.538381 | 0.0127 | up | 8.529071 |
| PIM3 | 61.5512 | 7.33083 | 0.00005 | up | 8.396212 |
| GDNF | 18.2254 | 2.24611 | 0.00065 | up | 8.114206 |
| SEMA4D | 5.63233 | 0.720616 | 0.00155 | up | 7.815994 |
| MSX1 | 45.7906 | 5.95609 | 0.00035 | up | 7.68803 |
| RUNX3 | 2.2623 | 0.304667 | 0.0337 | up | 7.425484 |
| HSPA1B | 59.3396 | 8.41269 | 0.00005 | up | 7.053582 |
| IFIH1 | 4.42257 | 0.694008 | 0.0309 | up | 6.372506 |
| FOXC2 | 16.1969 | 3.06861 | 0.00155 | up | 5.278253 |
| MX1 | 32.1865 | 6.17649 | 0.0021 | up | 5.211131 |
| ALDH1A3 | 19.0468 | 3.67015 | 0.03105 | up | 5.189652 |
| SOX9 | 8.88515 | 1.95518 | 0.0072 | up | 4.544415 |
| NOTCH1 | 11.5316 | 2.74678 | 0.0011 | up | 4.198225 |
| PRKCZ | 3.13343 | 0.853712 | 0.03545 | up | 3.67036 |

| | | | | | |
|---|---|---|---|---|---|
| ERN1 | 15.8008 | 4.44049 | 0.01565 | up | 3.558346 |
| HSPA1A | 61.4519 | 17.5863 | 0.00125 | up | 3.494305 |
| FOXC1 | 15.0835 | 4.4641 | 0.01085 | up | 3.378845 |
| ALB | 6.31432 | 1.98867 | 0.03395 | up | 3.175147 |
| GLI3 | 2.61378 | 0.856528 | 0.04375 | up | 3.051599 |
| SOCS3 | 36.4412 | 12.8045 | 0.01075 | up | 2.845968 |
| VEGFA | 38.8331 | 14.0268 | 0.00875 | up | 2.768493 |
| TNFRSF10D | 141.519 | 54.0409 | 0.0093 | up | 2.618739 |
| PMAIP1 | 40.1004 | 16.0506 | 0.03645 | up | 2.498374 |
| TLR4 | 3.29112 | 9.34058 | 0.03035 | down | 2.838116 |
| MAPK9 | 5.85134 | 13.993 | 0.0475 | down | 2.391418 |
| GAS1 | 8.53322 | 20.3735 | 0.04705 | down | 2.387551 |

**Table S1. Lists of differentially expressed genes in BJ cells at 6 hours after HSV-1 infection a.** Gene Ontology analysis of differentially expressed genes involved in transcription regulation, **b.** neuronal differentiation, **c.** immune system development and **d.** programmed cell death regulation after HSV-1 infection are listed here. “#DIV/0!” denotes infinite. “up” denotes up-regulation. “down” denotes down-regulation.

**Table S2. The comparison of RNA-seq data with microarray analysis from Pasieka et al., (ref 14)**

**Table S2a.** Genes involved in immune response

| mouse MEF | | human BJ | | | | | type |
|---|---|---|---|---|---|---|---|
| **Gene** | **fold** | **Gene** | **infection** | **control** | **fold** | **P_value** | |
| | | IFI44L | 3.44062 | 0.47809 | 7.196595 | P≤0.05 | ● |
| | | IL16 | 0.721267 | 0 | #DIV/0! | P≤0.05 | ● |
| | | IRF4 | 11.7536 | 0 | #DIV/0! | P≤0.05 | ● |
| | | IRF5 | 0.718981 | 0.041102 | 17.49256 | P≤0.05 | ● |
| | | MX1 | 32.1865 | 6.17649 | 5.211131 | P≤0.05 | ● |
| | | S100A8 | 0.771023 | 0 | #DIV/0! | P≤0.05 | ● |
| | | SOCS3 | 36.4412 | 12.8045 | 2.845968 | P≤0.05 | ● |
| | | STAT4 | 1.79078 | 0.375829 | 4.76488 | P≤0.05 | ● |
| Ifih1 | 15.1 | IFIH1 | 4.42257 | 0.694008 | 6.372506 | P≤0.05 | ※ |
| Ifit2 | 11.8 | IFIT2 | 38.1518 | 3.74599 | 10.1847 | P≤0.05 | ※ |
| Irf1 | 5.3 | IRF1 | 19.9638 | 6.21186 | 3.21382 | P≤0.05 | ※ |
| Irf7 | 8.6 | IRF7 | 11.0408 | 3.06429 | 3.603053 | P≤0.05 | ※ |
| Mx2 | 34.5 | MX2 | 8.23775 | 1.25839 | 6.546261 | P≤0.05 | ※ |
| Oasl1 | 11 | OASL | 11.9408 | 0.313339 | 38.10825 | P≤0.05 | ※ |
| Vegfa | 4.5 | VEGFA | 38.8331 | 14.0268 | 2.768493 | P≤0.05 | ※ |
| Ccl4 | 3.5 | CCL4 | 0.154648 | 0 | #DIV/0! | P≥0.05 | ※ |
| Ccl5 | 4.8 | CCL5 | 5.7192 | 0.848958 | 6.736729 | P≥0.05 | ※ |
| Cxcl10 | 96 | CXCL10 | 4.2195 | 0.640209 | 6.590816 | P≥0.05 | ※ |
| Cxcl11 | 12.5 | CXCL11 | 1.30639 | 0 | #DIV/0! | P≥0.05 | ※ |
| G1p2/ISG15 | 23.9 | ISG15 | 118.939 | 57.2818 | 2.076384 | P≥0.05 | ※ |
| Gbp4 | 27.5 | GBP4 | 2.59105 | 1.17546 | 2.204286 | P≥0.05 | ※ |
| Gbp5 | 8.6 | GBP5 | 0.063989 | 0.023132 | 2.766227 | P≥0.05 | ※ |
| Ifi1/IRGM | 14.7 | IRGM | 0 | 0 | | | ※ |
| Ifi44 | 15.6 | IFI44 | 14.5603 | 4.50861 | 3.229443 | P≥0.05 | ※ |
| Ifit1 | 36.8 | IFIT1 | 50.9492 | 9.62708 | 5.29228 | P≥0.05 | ※ |
| Ifit3 | 11.9 | IFIT3 | 13.1296 | 2.11088 | 4.319568 | P≥0.05 | ※ |
| S100a9 | 2.1 | S100A9 | 0.469722 | 0 | #DIV/0! | P≥0.05 | ※ |
| Socs1 | 4.8 | SOCS1 | 24.705 | 10.7602 | 2.295961 | P≥0.05 | ※ |
| Stat2 | 7.9 | STAT2 | 35.2059 | 26.6048 | 1.323291 | P≥0.05 | ※ |
| Vav2 | 15.2 | VAV2 | 24.2649 | 10.1929 | 2.380569 | P≥0.05 | ※ |
| Il15 | 3.5 | IL15 | 1.19395 | 2.78725 | 0.428361 | P≥0.05 | ※ |
| Irg1 | 8.5 | IRG1 | 0 | 0 | | | ★ |
| Npy | 8.1 | NPY | 0 | 0 | | | ★ |
| Ifnb | 3.2 | IFNB1 | 0 | 0 | | | ★ |
| Igtp | 23.6 | | No existence | | | | ★ |
| Iigp1 | 68.9 | | No existence | | | | ★ |
| Iigp2 | 15.9 | | No existence | | | | ★ |

**Table S2b.** Cytokine genes

| mouse MEF | | human BJ | | | | | type |
|---|---|---|---|---|---|---|---|
| Gene | fold | Gene | infection | control | fold | P_value | |
| Arc | 13.5 | ARC | 49.0085 | 0.192227 | 254.9512 | P≤0.05 | ※ |
| | | LGALS9 | 19.676 | 3.87434 | 5.078542 | P≤0.05 | ● |

**Table S2c.** Genes involved in cell death, cell-to-cell signaling

| mouse MEF | | human BJ | | | | | |
|---|---|---|---|---|---|---|---|
| Gene | fold | Gene | infection | control | fold | P_value | type |
| | | TNFRSF10D | 141.519 | 54.0409 | 2.618739 | P≤0.05 | ● |
| | | TNFAIP2 | 64.2931 | 19.338 | 3.324703 | P≤0.05 | ● |
| | | TLR4 | 3.29112 | 9.34058 | 0.352346 | P≤0.05 | ● |
| | | SERPINC1 | 0.779692 | 0 | #DIV/0! | P≤0.05 | ● |
| | | IRF7 | 11.0408 | 3.06429 | 3.603053 | P≤0.05 | ● |
| | | IRF1 | 19.9638 | 6.21186 | 3.21382 | P≤0.05 | ● |
| | | IL16 | 0.721267 | 0 | #DIV/0! | P≤0.05 | ● |
| | | IFI44L | 3.44062 | 0.47809 | 7.196595 | P≤0.05 | ● |
| | | ICAM5 | 19.5184 | 4.1356 | 4.719605 | P≤0.05 | ● |
| | | CD53 | 1.29916 | 0 | #DIV/0! | P≤0.05 | ● |
| Tnfsf10 | 2.2 | TNFSF10 | 0.873211 | 0 | #DIV/0! | P≤0.05 | ※ |
| Tnf | 2.38 | TNF | 0.266176 | 0.050315 | 5.290234 | P≥0.05 | ※ |
| Tlr3 | 2.26 | TLR3 | 0.52493 | 0.323608 | 1.622117 | P≥0.05 | ※ |
| Notch1 | 1.24 | NOTCH1 | 11.5316 | 2.74678 | 4.198225 | P≤0.05 | ※ |
| Ifi4 | 7.02 | IFI4 | 9.28068 | 0.633051 | 14.66024 | P≤0.05 | ※ |
| Ccl4 | 3.48 | CCL4 | 0.154648 | 0 | #DIV/0! | P≥0.05 | ※ |
| Casp1 | 1.84 | CASP1 | 4.82005 | 2.73361 | 1.763254 | P≥0.05 | ※ |
| B2m | 1.5 | B2M | 2866.35 | 2030.18 | 1.41187 | P≥0.05 | ※ |
| Tlr2 | 1.44 | TLR2 | 0.054963 | 0.607747 | 0.090437 | P≥0.05 | ★ |
| Serpine1 | 1.01 | SERPINE1 | 93.7985 | 119.394 | 0.785622 | P≥0.05 | ★ |
| Prkr | 4.13 | EIF2AK2 | 12.1812 | 14.9882 | 0.812719 | P≥0.05 | ★ |
| Nfkb1 | 1.51 | NFKB1 | 11.9157 | 13.3461 | 0.892823 | P≥0.05 | ★ |
| Myc | 1.41 | MYC | 12.7983 | 16.7395 | 0.764557 | P≥0.05 | ★ |
| Il15 | 3.48 | IL15 | 1.19395 | 2.78725 | 0.428361 | P≥0.05 | ★ |
| Icam1 | 1.68 | ICAM1 | 4.87759 | 5.80746 | 0.839884 | P≥0.05 | ★ |
| Hck | 1.15 | HCK | 0 | 0.017087 | 0 | P≥0.05 | ★ |
| Cxcl9 | 8.54 | CXCL9 | 0 | 0.035336 | 0 | P≥0.05 | ★ |
| Cd47 | 1.3 | CD47 | 22.0311 | 30.3119 | 0.726814 | P≥0.05 | ★ |
| Ccl7 | 2.87 | CCL7 | 3.66504 | 5.29228 | 0.692526 | P≥0.05 | ★ |
| Ccl3 | 1.6 | CCL3 | 0 | 0 | | | ★ |
| Ifnb | 3.22 | IFNB | 0 | 0 | | | ★ |
| Cd69 | 4.52 | CD69 | 0 | 0 | | | ★ |
| Ccl12 | 1.42 | | No existence | | | | ★ |
| Ifi202 | 5.16 | | No existence | | | | ★ |

**Table S2. The comparison of RNA-seq data with microarray analysis from Pasieka et al., (ref 14) a.** Compared to microarray data, RNA-seq showed the difference of the expression of some genes involved in immune response, **b.** cytokine and **c.** cell death, cell-to-cell signaling between control and HSV-1 infection: “●” denotes some genes not reported previously; “※” demotes some genes were recovered in RNA-seq data; “★” denotes some genes were not significantly affected by HSV-1 infection based on RNA-seq analysis. “#DIV/0!” denotes infinite. “No existence” showed there is no corresponding gene in human genome.

**Table S3. Sequences of primers used for RT-qPCR or RT-PCR**

**Table S3a. Sequences of primers used for RT-qPCR**

| primers | sequences |
|---|---|
| 18S-F | 5’ GTAACCCGTTGAACCCCATT 3’ |
| 18S-R | 5’ CCATCCAATCGGTAGTAGCG 3’ |
| COMP-F: | 5’ CCTGGGGGTCTTCTGCTT 3’ |
| COMP-R: | 5’ TCCTCTGGGATGGTGTCATT 3’ |
| IFIH1-F | 5’ ACCAAATACAGGAGCCATGC 3’ |
| IFIH1-R | 5’ GCGATTTCCTTCTTTTGCAG 3’ |
| H2AFX-F | 5’ TACCTCACCGCTGAGATCCT 3’ |
| H2AFX-R | 5’ CTGGATGTTGGGCAGGAC 3’ |
| OAS1-F | 5’ CAAGCTCAAGAGCCTCATCC 3’ |
| OAS1-R | 5’ TGGGCTGTGTTGAAATGTGT 3’ |
| CBX4-F: | 5’ GTGAAATGGAGAGGCTGGTC 3’ |
| CBX4-R: | 5’ CTGCCGTTCCCTGTTCTG 3’ |
| BRD2-F: | 5’ CCATCTTGGCTCCTGGTTC 3’ |
| BRD2-R: | 5’ GTGCTGCCTTAGGCTCAAGA 3’ |
| GTF2F1-F | 5’ ACGTCGTTCGAGTTCCTAAGA 3’ |
| GTF2F1-R | 5’ GATTCCACGTAGCAAAGTTGACT 3’ |
| MX2-F | 5’ CTGGAGGCACTGTCAGGAGT 3’ |
| MX2-R | 5’ CGGACACCTGGTTACGATTC 3’ |
| DDX58-F | 5’ GAGGCTGCCACACTTTTTCT 3’ |
| DDX58-R | 5’ GGCTTCATAAAGTCCAGAATAACC 3’ |
| HERC5-F | 5’ CACAGAATGAGCTAAGACCCTGT 3’ |
| HERC5-R | 5’ AGAAACATAGGCAAGTGTGTGC 3’ |
| GAS1-F | 5’ TCTCGACAGCTGTTCATTTCC 3’ |
| GAS1-R | 5’ GCAGAAGGTCCCCTTTCG 3’ |
| TLR4-F | 5’ GTCTGCAGGCGTTTTCTTCT 3’ |
| TLR4-R | 5’ AAGTGAAAGCGGCAACCTTA 3’ |
| H19-F: | 5’ GTACAGCATCCAGGGGAGTC 3’ |
| H19-R: | 5’ TCCCTCCTGAGAGCTCATTC 3’ |
| MEG9-F: | 5’ TAGGGTCGGTCTTGAGAAGG 3’ |
| MEG9-R: | 5’ GAGCGAGGAGCTACACCAAA 3’ |

| | |
|---|---|
| BCL2L11-F<br>BCL2L11-R | 5’ TAAGTTCTGAGTGTGACCGAGA 3’<br>5’ GCTCTGTCTGTAGGGAGGTAGG 3’ |
| PAPD7-F<br>PAPD7-R | 5’ AAGCAGGACTGGCTCACTCT 3’<br>5’ TGGAATGCCTCCTAGAGAACC 3’ |
| CSRNP2-F<br>CSRNP2-R | 5’ AAAGCCTTTTCTGGTGGGATA 3’<br>5’ TGCGACATACGTGATGGTTT 3’ |
| TFAP2A-F<br>TFAP2A-R | 5’ TCCATGCCTGTCAATGTATCA 3’<br>5’ AAAATCGACATAAAGCGTATCAAA 3’ |
| NIT1-F<br>NIT1-R | 5’ ATGTCAAACATGCGGTGCAG 3’<br>5’ TGCCCTTGAGTGTCTCATCG 3’ |
| RUNX1-F<br>RUNX1-R | 5’ CTCTGTGTGCCTCAGTGGAG 3’<br>5’ CAGCCTGGTGAAAGCAACAC 3’ |
| SREK1-F<br>SREK1-R | 5’ ACCGACAAGTGTACCTCTGC 3’<br>5’ CAGCCACCATGGAAATGCAC 3’ |
| ABCC5-F<br>ABCC5-R | 5’ GGGGAGTTTCTTTCCTGCCTTCT 3’<br>5’ TCCTGAGCCCTGCCACTGAAC 3’ |
| IGF2-F<br>IGF2-R | 5’ GCCATTCGGAACATTGGACAG 3’<br>5’ CACGACTAGGCACGGAGGTCA 3’ |
| SNHG9-F<br>SNHG9-R | 5’ GCGTCTCGCTGGTGGTCTTCG 3’<br>5’ CCTGTCGGGGCCTGGGTTTCA 3’ |
| KLHL21-F<br>KLHL21-R | 5’TCCAAACCTACACTCCGTGAC 3’<br>5’TTTCCCAAATTCGCTATGACTA 3’ |
| DGAT1-2-F<br>DGAT1-2-R | 5’ TGGTGCATCAGGTAGG 3’<br>5’ TGGGCTTGTAGAAGTGTC 3’ |
| DGAT1-1-F<br>DGAT1-1-R | 5’ CTGACGGAGCAGGCGGGA 3’<br>5’ CTGGAGTGATAGACTCAA 3’ |

**Table S3b. Sequences of primers used for RT-PCR**

| primers | sequences |
|---|---|
| HCFC1R1-F<br>HCFC1R1-R | 5’ GAGAGGGGAGCTGGGAAA 3’<br>5’ GCCTTGGGGGTGACTTGG 3’ |
| STK11-F<br>STK11-R | 5’ AAGGGGACAACATCTACA 3’<br>5’ GATGGAGAACCTCTTGGC 3’ |
| c-FOS-F<br>c-FOS-R | 5’ AGAATCCGAAGGGAAAGG 3’<br>5’ GCGAGTCAGAGGAAGGCT 3’ |

| | |
|---|---|
| NUFIP2-F<br>NUFIP2-R | 5’ AGAATGCTACCCAGAACTT 3’<br>5’ CTGCTAATACTCTAACACCC 3’ |
| SRSF6-F<br>SRSF6-R | 5’ TGAGGCTAAGATGACTGC 3’<br>5’ GTGAGGTTTTTGGTGGTG 3’ |
| PAPD7-F<br>PAPD7-R | 5’ TTGAGGTAGCCGTGTCTGT 3’<br>5’ TGGTTTCCTTAATGTGGGT 3’ |
| CSRNP2-F<br>CSRNP2-R | 5’ GAGGAGGGAGTGGAAGGGTC 3’<br>5’ AAGGGTTGTGGTAATAAGAGT 3’ |
| TFAP2A-F<br>TFAP2A-R | 5’GGACTGAGTCACCACCTTC 3’<br>5’TCTAAATGATACCCTGCTC 3’ |
| NIT1-F<br>NIT1-R | 5’ GCAGGTCGTGGCGAGTGATGAT 3’<br>5’ AGCAGGGATTCTGGAAAGCATT 3’ |
| RUNX1-F<br>RUNX1-R | 5’ AGTTCCAGAGGGTTGAGGCAGG 3’<br>5’ CTGGGTGGGGGTATGTGCTATC 3’ |
| SREK1-F<br>SREK1-R | 5’ GATCCAAGTTTGTACTATCCCTA 3’<br>5’ GTTTACCAGCCAACCGTAT 3’ |
| ABCC5-F<br>ABCC5-R | 5’ GGGGAGTTTCTTTCCTGCCTTCT 3’<br>5’ TCCTGAGCCCTGCCACTGAAC 3’ |
| IGF2-F<br>IGF2-R | 5’ GCCATTCGGAACATTGGACAG 3’<br>5’ CACGACTAGGCACGGAGGTCA 3’ |
| STK11-control F<br>STK11-control R | 5’ AGCCGGGACTGACGTGTA 3’<br>5’ TGGCGTTTCTCGTGTTTT 3’ |
| c-FOS-control F<br>c-FOS-control R | 5’ GAAGCCAAGACTGAGCCG 3’<br>5’ CAGCACCAGGTTAATTCCAAT 3’ |
| NUFIP2-control F<br>NUFIP2-control R | 5’ GTCCCAGCCGCTTTCAAT 3’<br>5’ ATGGTTCCTCTGCTGCGTTGA 3’ |
| SNHG9-F<br>SNHG9-R | 5’ GCGTCTCGCTGGTGGTCTTCG 3’<br>5’ CCTGTCGGGGCCTGGGTTTCA 3’ |
| KLHL21-F<br>KLHL21-R | 5’TCCAAACCTACACTCCGTGAC 3’<br>5’TTTCCCAAATTCGCTATGACTA 3’ |
| DGAT1-2-F<br>DGAT1-2-R | 5’ TGGTGCATCAGGTAGG 3’<br>5’ TGGGCTTGTAGAAGTGTC 3’ |
| DGAT1-1-F<br>DGAT1-1-R | 5’ CTGACGGAGCAGGCGGGA 3’<br>5’ CTGGAGTGATAGACTCAA 3’ |

## Table S4. Gene displaying change of APA after HSV-1 infection

**Table S4a.** Genes involved in transcriptional regulation

| gene | adjusted.*P*_val | lengthening/shortening |
|---|---|---|
| SMARCA4 | 0.049211 | shortening |
| RELB | 0.04038 | shortening |
| HIRA | 0.036968 | shortening |
| MAFK | 0.018752 | shortening |
| SMARCC2 | 0.017756 | shortening |
| SLC30A9 | 0.016825 | shortening |
| PRDM4 | 0.016392 | shortening |
| SMARCD2 | 0.007408 | shortening |
| DDIT3 | 0.00376 | shortening |
| ARID3A | 0.002684 | shortening |
| TFAP2A | 0.0026 | shortening |
| RYBP | 0.001833 | shortening |
| CSRNP2 | 0.000806 | shortening |
| FAM120B | 0.000176 | shortening |
| BAZ2A | 0.000103 | shortening |
| NR2F1 | 5.22E-05 | shortening |
| USP16 | 7.19E-06 | shortening |
| BRE | 1.94E-06 | shortening |
| UBP1 | 5.65E-07 | shortening |
| TBX5 | 3.69E-07 | shortening |
| ECD | 3.46E-07 | shortening |
| TSC22D4 | 9.63E-08 | shortening |
| PAPD7 | 8.05E-08 | shortening |
| SSRP1 | 7.6E-08 | shortening |
| EHMT2 | 4.69E-08 | shortening |
| RUNX1 | 7.28E-09 | shortening |
| TBX3 | 7.01E-10 | shortening |
| GATA2 | 1.13E-17 | shortening |
| H2AFV | 0.041968 | lengthening |
| ATXN7L3 | 0.007706 | lengthening |
| HNRPDL | 0.000405 | lengthening |
| SMARCA1 | 1.97E-11 | lengthening |

**Table S4b.** Genes involved in stress response

| gene | adjusted.*P*_val | lengthening/shortening |
|---|---|---|
| LOC100133091 | 0.024308 | shortening |
| DDIT3 | 0.00376 | shortening |
| PCBP4 | 0.000252 | shortening |
| PPP1R15A | 0.000212 | shortening |
| ARFGAP3 | 3.24E-06 | shortening |
| BRE | 1.94E-06 | shortening |
| PAPD7 | 8.05E-08 | shortening |
| PAPD7 | 8.05E-08 | shortening |
| SSRP1 | 7.6E-08 | shortening |
| ACAP3 | 0.005659 | lengthening |
| CSNK1D | 1.06E-07 | lengthening |
| IGF2 | 1.01E-09 | lengthening |
| INS-IGF2 | 6.47E-10 | lengthening |

**Table S4c.** Genes involved in metabolic processes

| gene | adjusted.*P*_val | lengthening/shortening |
|---|---|---|
| SMARCC2 | 0.017756 | shortening |
| DDIT3 | 0.00376 | shortening |
| CSRNP2 | 0.000806 | shortening |
| USP16 | 7.19E-06 | shortening |
| BRE | 1.94E-06 | shortening |
| TBX5 | 3.69E-07 | shortening |
| RUNX1 | 7.28E-09 | shortening |
| TBX3 | 7.01E-10 | shortening |
| GATA2 | 1.13E-17 | shortening |
| ATXN7L3 | 0.007706 | lengthening |
| IGF2 | 1.01E-09 | lengthening |
| INS-IGF2 | 6.47E-10 | lengthening |
| SMARCA1 | 1.97E-11 | lengthening |

**Table S4d.** Genes involved in nuclear export

| gene | adjusted.*P*_val | lengthening/shortening |
|---|---|---|
| SEC24D | 0.006595 | lengthening |
| MRPL45 | 8.22E-06 | shortening |
| PEX6 | 0.000813 | lengthening |
| GSK3B | 0.000289 | shortening |
| SMG7 | 8.22E-06 | shortening |
| THOC7 | 0.0132 | lengthening |
| TOM1 | 0.011204 | lengthening |
| SNX9 | 0.018829 | lengthening |
| M6PR | 0.016816 | shortening |
| GLE1 | 0.000475 | shortening |
| ARL1 | 7.48E-05 | lengthening |
| ARFGAP3 | 3.24E-06 | shortening |
| AP1M1 | 2.24E-05 | shortening |

**Table S4. Gene displaying changes of APA after HSV-1 infection.** Genes showing changes in APA after HSV-1 infection are listed according to GO terms: **a.** Transcriptional regulation, **b.** metabolic process, **c.** metabolic process and **d.** nuclear export.

**Table S5. Cell cycle genes exhibiting AS**

| Term | Count | PValue | Genes |
|---|---|---|---|
| cell cycle | 66 | 1.08E-06 | TUBB2A, AURKA, RBM7, PTTG1, SART1, CUL2, MCM7, PCBP4, PSMC3IP, PSMD3, CDK10, DNAJC2, ANAPC2, CHTF8, PAPD5, RAD52, RAD1, REC8, SGSM3, CDK11A, TBRG1, CLIP1, HAUS7, MAPK7, AKAP8, SEPT6, CHFR, SEPT8, SEPT9, MAP3K11, BCAT1, ZMYND11, PARD3, STK11, PML, DMWD, CHEK1, CHEK2, RCC1, SESN1, TCF7L2, PSMB6, MACF1, SAC3D1, SMARCB1, RB1CC1, TFDP2, PPP3CB, PKD1, PBRM1, NFATC1, GPS1, NASP, PSRC1, WDR6, STRADA, CENPE, CEP63, CDC27, CDC25B, CDKN1C, MAPK12, CUL4B, ARAP1, TXNL4A, DNM2 |
| cell cycle process | 49 | 2.04E-05 | BCAT1, TUBB2A, STK11, PML, CHEK1, DMWD, AURKA, RBM7, PTTG1, RCC1, TCF7L2, SESN1, SART1, CUL2, PSMB6, MACF1, SAC3D1, PCBP4, PSMC3IP, PPP3CB, PSMD3, PKD1, PBRM1, CDK10, DNAJC2, NFATC1, ANAPC2, WDR6, CENPE, PAPD5, RAD52, CEP63, CDC27, CDC25B, RAD1, CDKN1C, REC8, MAPK12, SGSM3, CDK11A, TBRG1, CLIP1, HAUS7, AKAP8, CHFR, ARAP1, TXNL4A, MAP3K11, DNM2 |
| cell cycle arrest | 14 | 0.000763 | STK11, WDR6, PML, SESN1, TCF7L2, SART1, CDKN1C, CUL2, MAPK12, MACF1, SGSM3, PCBP4, TBRG1, PKD1 |
| cell cycle phase | 34 | 0.001209 | BCAT1, TUBB2A, CHEK1, DMWD, RBM7, AURKA, PTTG1, RCC1, CUL2, SAC3D1, PSMC3IP, PPP3CB, PBRM1, CDK10, DNAJC2, NFATC1, ANAPC2, CENPE, PAPD5, CEP63, RAD52, CDC27, CDC25B, RAD1, CDKN1C, REC8, CDK11A, CLIP1, HAUS7, AKAP8, CHFR, TXNL4A, MAP3K11, DNM2 |

**Table S5. Cell cycle genes exhibiting AS:** Cell cycle related genes that underwent AS after HSV-1 infection.

**Table S6.** Immunity and apoptotic genes that underwent AS, APA and isoform switches

**S6a.** Immunity genes that underwent AS, APA and isoform switches

| **ISO** | **DE** |
|---|---|
| XRCC6,TTC7A,TP53,SP1,SMAD5,RELB,PSEN1,PBX1,NKX2-3,MLL5,MAEA,LIG4,JMJD6,JAG1,IRF4, IRF1,IFI16,HOXA9,FOXP1,FLT3LG,EGR1, DYRK3,CDK6,BCL2L11,BARX1, | NKX2-3,MLL5,JAG1,IRF4,IRF1,HOXA9, EGR1,BCL2L11,BARX1,ACE,VEGFA,TBX1, PLCG2,KITLG,HOXB4,HOXB3,HOXA3, |

**S6b.**Apoptotic genes that underwent AS, APA and isoform switches

| ISO | AS | APA |
|---|---|---|
| MAEA,MITF,CBX4, FOXO1,PMAIP1,GDNF, GLI3,SHH,CUL5, ATG5,CASP8AP2, CHST11,MX1,CASP2, DEDD2,CUL1,FGF4, IHH,ARHGEF4, ARHGEF3,CRYAA, NKX2-6,SOCS3, TP53,ADNP,ACTN1, PIM3,MBD4,POLB, IFI16,LIG4,PRKCE, STK4,BCL2L11,MSX1, PSEN1,RASGRF2, TNFRSF10D,ERN1, MAPK9,FOXC2,FOXC1, GSTP1,ACVR1,BID, IFIH1,BCLAF1,PAFAH2, ASNS,HSPA1A,HSPA1B, NR3C1,CALR,SOX9, TCF7L2,PEA15,IGF1R, ALB,ALDH1A3,NPM1, PLEKHG5,TNFRSF19, POU3F3,ERCC3, THBS1,NEFL,HIP1, MUC2,HTT,IGF2GAS1 YWHAESIRT1,PPIF,CDH13, NOTCH1,DLX1,GSPT1,BNIP3L, MUC5AC,IKBKB,APBB2,IGFBP3 | DEDD,FASTK,RBM5, EIF5A,PRDX2,CIAPIN1, SART1,RPS3,CUL2, CASP3,BDNF,CD44, PCBP4,NQO1,NRG1, IP6K2,IRAK1,IFI16, ARHGEF12,ECT2, BCL2L11,TIAL1,HIPK2, KALRN,MAP3K11, HMGB1,CDK5R1,SYVN1, GCLC,TNFRSF25, ADAMTSL4,PML,CHEK2, TCF7L2,ADA,PLEKHG2, MOAP1,COMP,RB1CC1, TRAF3,HSPA9,CARD8, TBX3,CREB1,SMAD6, SPHK1,IGF2,SAP30BP, SON,EI24,PDCD5,DNM2, | PTPRF,TBX3,INS-IGF2, TBX5,ARHGEF17,IGF2, ANXA5,DDIT3,PLAGL1, FNTA,PCBP4,GSK3B, SERPINB2,CAT,TIAF1, DNAJB6,<br>DE<br>GLI3, IHH, NKX2-6, PIM3 TNFRSF10D,MAPK9, IFIH1, ALB, POU3F3, MUC2, NOTCH1,MUC5AC, PMAIP1,SHH,MX1,CRYAA, SOCS3, BCL2L11, ERN1,FOXC2, HSPA1A,ALDH1A3, DLX1, CBX4, FGF4,FOXC1, GDNF, HSPA1B,MSX1, SOX9 |

**Table S6. Immunity genes and apoptotic genes that underwent AS, APA and isoform switches. a.** Immunity genes that underwent AS, APA and isoform switches. **b.** Apoptotic genes that underwent AS, APA and isoform switches. Genes involved in apoptotic process that are exhibiting changes in isoform, AS and APA changes after HSV-1 infection are listed. Apoptotic genes that underwent differential expression are listed in Table S1d.

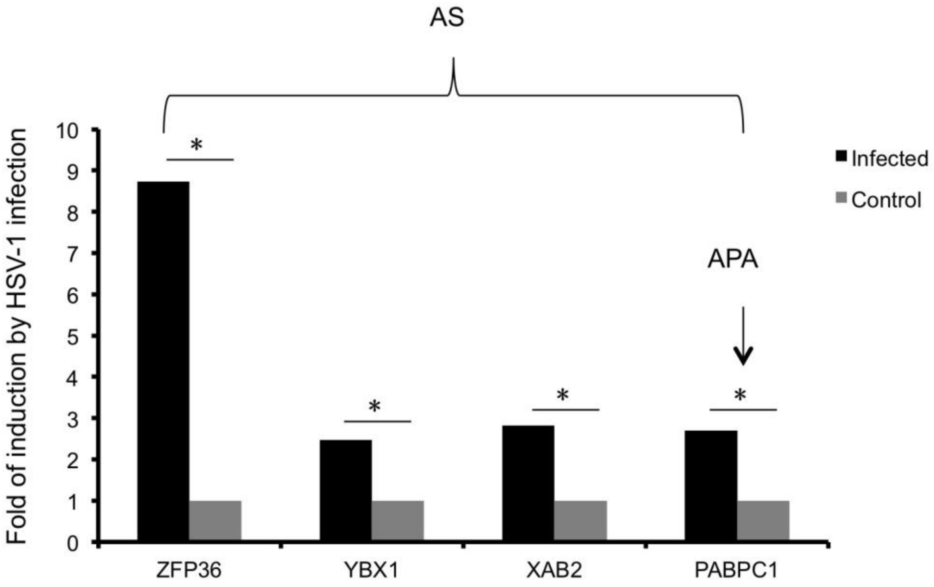


**Figure S1. Examples of up regulated genes implicated in RNA splicing after HSV-1 infection.**

Gene Ontology analysis showed that PABPC1, YBX1, ZFP36 and XAB2 that were up regulated 2 fold or more after HSV-1 infection. * denotes significance, *p* value <0.05.
